# Computational analysis of the effects of geometric irregularities and post-processing steps on the mechanical behavior of additively manufactured 316L stainless steel stents

**DOI:** 10.1371/journal.pone.0244463

**Published:** 2020-12-29

**Authors:** Lisa Wiesent, Ulrich Schultheiß, Philipp Lulla, Ulf Noster, Thomas Schratzenstaller, Christof Schmid, Aida Nonn, Ashley Spear

**Affiliations:** 1 Computational Mechanics and Materials Lab, Department of Mechanical Engineering, Ostbayerische Technische Hochschule (OTH) Regensburg, Regensburg, Germany; 2 Technology Campus Neustadt a. d. Donau, Department of Mechanical Engineering, OTH Regensburg, Regensburg, Germany; 3 Material Science and Surface Analytics Lab, Department of Mechanical Engineering, OTH Regensburg, Regensburg, Germany; 4 FIT Production GmbH, Lupburg, Germany; 5 University Hospital Regensburg, University Regensburg, Regensburg, Germany; 6 Medical Device Lab, Department of Mechanical Engineering, OTH Regensburg, Regensburg, Germany; 7 Regensburg Center of Biomedical Engineering (RCBE), Regensburg, Germany; 8 Department of Mechanical Engineering, University of Utah, Salt Lake City, Utah, United States of America; University of Vigo, SPAIN

## Abstract

Advances in additive manufacturing enable the production of tailored lattice structures and thus, in principle, coronary stents. This study investigates the effects of process-related irregularities, heat and surface treatment on the morphology, mechanical response, and expansion behavior of 316L stainless steel stents produced by laser powder bed fusion and provides a methodological approach for their numerical evaluation. A combined experimental and computational framework is used, based on both actual and computationally reconstructed laser powder bed fused stents. Process-related morphological deviations between the as-designed and actual laser powder bed fused stents were observed, resulting in a diameter increase by a factor of 2-2.6 for the stents without surface treatment and 1.3-2 for the electropolished stent compared to the as-designed stent. Thus, due to the increased geometrically induced stiffness, the laser powder bed fused stents in the as-built (7.11 ± 0.63 N) or the heat treated condition (5.87 ± 0.49 N) showed increased radial forces when compressed between two plates. After electropolishing, the heat treated stents exhibited radial forces (2.38 ± 0.23 N) comparable to conventional metallic stents. The laser powder bed fused stents were further affected by the size effect, resulting in a reduced yield strength by 41% in the as-built and by 59% in the heat treated condition compared to the bulk material obtained from tensile tests. The presented numerical approach was successful in predicting the macroscopic mechanical response of the stents under compression. During deformation, increased stiffness and local stress concentration were observed within the laser powder bed fused stents. Subsequent numerical expansion analysis of the derived stent models within a previously verified numerical model of stent expansion showed that electropolished and heat treated laser powder bed fused stents can exhibit comparable expansion behavior to conventional stents. The findings from this work motivate future experimental/numerical studies to quantify threshold values of critical geometric irregularities, which could be used to establish design guidelines for laser powder bed fused stents/lattice structures.

## 1 Introduction

Advances in laser powder bed fusion (L-PBF) for metal additive manufacturing (AM) allow for the generation of highly porous cellular structures in the micrometer range, making this technology particularly well-suited for manufacturing custom lattice structures [1–3]. L-PBF is also commonly known as selective laser melting (SLM) or direct metal laser melting (DMLM) [4]. In the medical sector, AM lattice structures are considered for bone scaffolds and customized implants, e.g. orthopedic and dental implants [5, 6] and more recently for cardiovascular implants, so-called stents [7, 8]. Stents are used for the treatment of vascular constrictions in the course of coronary heart disease. Conventionally, stents are laser cut from small metal tubes [9]. The basic stent dimensions are thus determined by the tube, impeding the implementation of patient-specific designs or the consideration of arterial bifurcations. With AM, patient-specific stents could be realized that consider, for example, local diameter profiles, curvatures, or bifurcations, thus enabling a better adherence of the stent to the vascular wall. Balloon-expandable cardiovascular stents are commonly made from materials with high strength and corrosion resistance, such as stainless steel (316L) and cobalt chromium (CoCr) alloys [9].

Previous work on AM metallic cardiovascular stents is currently limited to feasibility studies regarding the possibility of producing expandable stent geometries using L-PBF [7, 10–12]. The generation of cardiovascular stents using L-PBF is a great challenge due to the small diameter of the stent struts (60-150 *μ*m), which is both comparable to the laser spot of industrial L-PBF machines and the powder size used (10-50 *μ*m) [7, 11]. In 2017, Demir et. al. [7] succeeded in producing the first CoCr stent prototypes via selective laser melting. In a follow-up study, Finazzi et al. [10] derived design guidelines for tubular and branched cardiovascular CoCr stents produced by L-PBF. Accordingly, due to the small structural size, no support structures can be used, and the stents must therefore be self-supporting (closed-cell design) [10]. Only recently, the principle functionality of L-PBF stents made from CoCr and a novel biodegradable FeMnCS alloy was demonstrated by means of their expandability using a balloon catheter [10, 12]. However, it was further found that L-PBF stents exhibited geometric and dimensional deviations compared to the intended stent, especially with respect to strut shape and strut thickness [10]. More specifically, the struts had a round rather than an intentionally rectangular cross-section and a strut thickness error of up to 75 *μ*m at an intended strut thickness of 120 *μ*m. In addition, mechanical testing of the L-PBF struts showed a lower Young’s modulus E ≈ 50 MPa compared to SLM CoCr bulk material (E = 210 MPa) [10]. In depth investigations of the mechanical behavior of SLM stents or the influence of the geometric irregularities have not yet been carried out, to the authors’ knowledge. However, such investigations are essential, since the mere proof of manufacturability and selective functionality of stents is not sufficient to exploit the advantages of AM in this specific medical field. Further, although the use of optimization tools to improve the expansion and fatigue behavior of L-PBF stents has been recommended [11], it has not yet been investigated whether the numerical tools commonly used for conventional stents are also suitable for L-PBF stents.

Due to the similar topology, the knowledge gained about the mechanical and morphological properties, as well as the computational analysis of L-PBF lattice structures, can also be considered for L-PBF stents. Design development or topological optimization of conventional stent is currently based on idealized computer aided design (CAD) models with homogeneous material properties [13–15]. However, studies on L-PBF lattice structures have shown that L-PBF leads to a variety of process-related deviations between the as-built and the intended as-designed morphologies. These are characterized by geometric irregularities, including local variations in strut diameter and cross-sectional shape, surface roughness, strut waviness, tapers, and porosity [16–20]. Combined experimental and numerical studies have further shown that these geometric irregularities have a distinct influence on the mechanical properties of L-PBF lattice structures, leading to differences between the numerical prediction based on the as-designed geometry and the experimentally determined mechanical response of the L-PBF structure [6, 16–20]. These studies suggest that reconstruction from, for example, X-ray computed tomography (CT) or statistical consideration of process-related geometric irregularities is necessary for accurate prediction of the process-structure-property relationship of L-PBF lattice structures. Conventional stent development/optimization approaches based on idealized models are, therefore, probably not suitable for L-PBF stents.

The mechanical behavior of L-PBF stents is further expected to be influenced by the size effect, resulting in deviation between the mechanical response of the bulk material and the actual L-PBF lattice structures [11, 19, 21, 22]. A preliminary study by the authors of this paper has shown that the yield strength of L-PBF 316L flat samples with a thickness of 0.5 mm is reduced by about 30% compared to samples with a thickness of 2 mm [23]. This trend is further supported by the findings of Karamooz Ravari et al. [19], which found a reduction in strength of L-PBF 316L lattice struts (200 *μ*m) by about 25% compared to L-PBF 316L bulk material.

Besides the geometric irregularities induced by L-PBF, the morphology and mechanical behavior of the stents are further influenced by post-processing steps, such as heat treatment or electropolishing. Electropolishing is intended to smooth the surface of the stents and to achieve an anti-corrosion passivization layer for the 316L [24, 25]. The microstructure and corresponding mechanical properties can also be modified by post-build heat treatments. Compared to L-PBF 316L in the as-built condition, a reduction in the yield and tensile strength can be achieved with a simultaneous increase in ductility due the homogenization of the microstructure (e.g. increase in grain size and misorientation), the reduction of the *δ*-ferrite phase and the reduction of residual stresses by adequate heat treatment [26–28].

In summary, geometric irregularities related to the L-PBF process, post-processing steps, and the size effect are all expected to have an impact on the mechanical response of L-PBF stents. The development and optimization of L-PBF stent designs based on idealized CAD models are, therefore, likely not appropriate. Thus, the applicability of the numerical methods established for the analysis and optimization of conventionally laser-cut stents should be investigated. In light of the above overview, the aim of this paper is to analyze the impact of process-induced geometric irregularities, the size effect, and post-processing steps on the mechanical response of L-PBF stents using a combined experimental and computational framework. Moreover, a numerical framework for the evaluation of both the mechanical properties and the expansion behavior of L-PBF stents should be provided and compared to existing approaches for conventionally manufactured stents.

Within the experimental framework, cardiovascular stents are produced from 316L stainless steel using L-PBF. Subsequently, the stents are selectively subjected to post-processing, targeting three different stent conditions: i) as-built, ii) heat treated, and iii) electropolished and heat treated condition. One representative stent per stent condition is analyzed by X-ray CT for morphological and subsequent numerical analysis. The compression behavior of the L-PBF stents is then evaluated using a compression test between two plates according to ISO 25539-2 [29]. To characterize the material behavior of L-PBF 316L, uniaxial tensile tests on flat specimens are performed, both in the as-built and heat treated condition.

Within the numerical framework, three representative stent models are reconstructed from the X-ray CT data: i) stent_AB_ in the as-built, ii) stent_HT_ in the heat treated, and iii) stent_EP-HT_ in the electropolished and heat treated condition. Flow curves are then derived from the uniaxial tensile tests to describe the material behavior of L-PBF 316L in the as-built and heat treated condition. Thereupon, the three reconstructed stent models are analyzed using finite element analysis (FEA). Two loading scenarios are simulated: i) compression between parallel plates and ii) stent crimping and free expansion. The numerical study is further extended to include the idealized CAD model stent_CAD_ on which the L-PBF stents are based, which allows for the comparison between the actual and as-designed mechanical response of the stents. The numerical predictions are then validated against the experimental data before the influence of post-processing (heat treatment, surface treatment) on the development of the stress states within the stent are evaluated based on the numerical analyses.

## 2 Methods

### 2.1 Design of the stent

**Fig 1:**
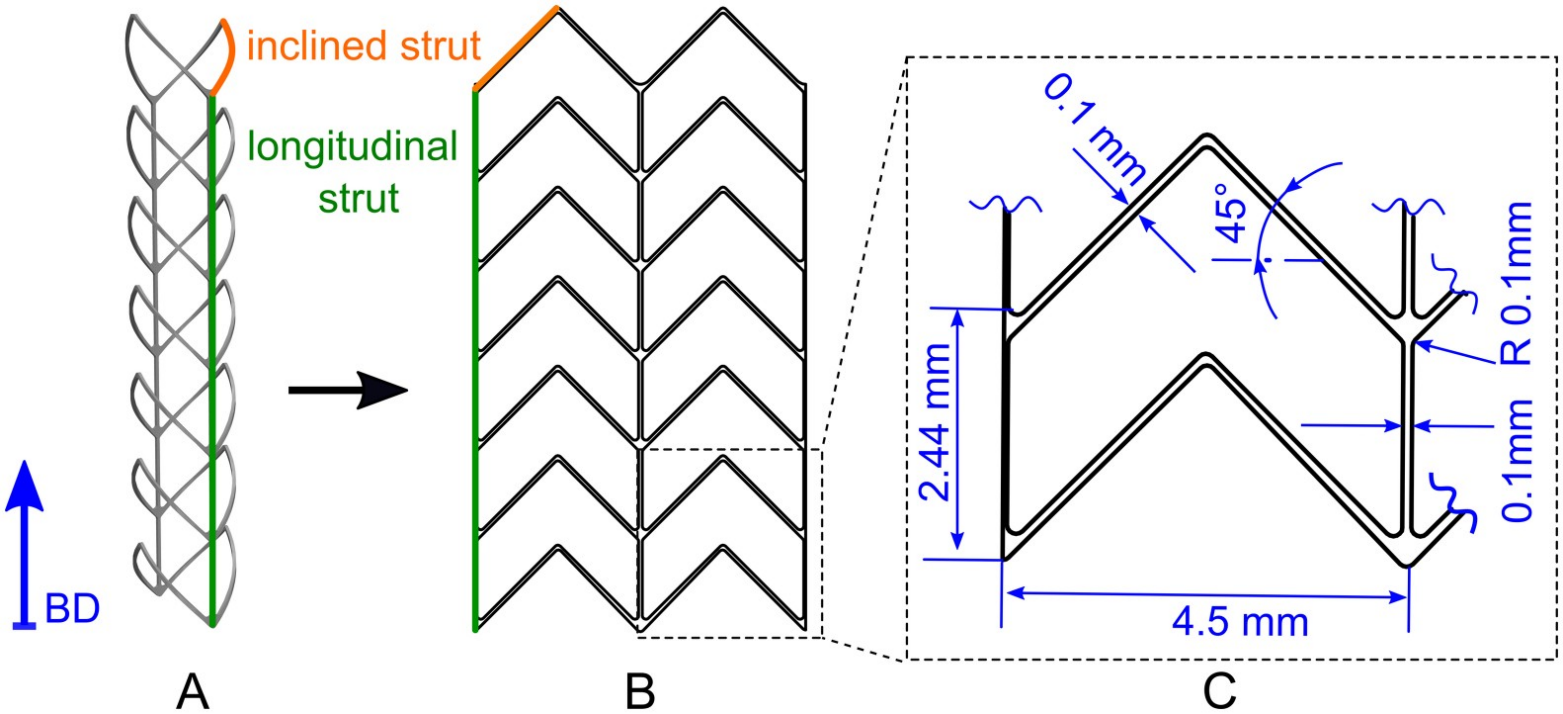
Intended stent design. A: stent CAD model, B: flat projection of the stent CAD model and C: basic dimension of the intended stent illustrated within the magnified stent unit cell. The later built direction (BD) within the laser powder bed fusion process is indicated by the blue arrow.

The L-PBF stents have a self-supporting, closed-cell design composed of two unit cells in the circumferential direction and six unit cells in the axial direction. The unit cell exhibits a zigzag pattern of serrated main struts (spikes) with an angle of 45° (Fig 1, orange) and two non-flexible connecting struts along the longitudinal stent axis (Fig 1, green). The stent struts have a quadratic cross-section with a thickness of 0.1 mm. The stents have a length of 16.94 mm and a nominal outer diameter of 3 mm. After stent expansion, a nominal outer stent diameter of 3.7 mm is intended. The stent design and its basic dimensions are illustrated in Fig 1. The stent design was chosen so that it can be manufactured without support structures using L-PBF. It is not intended to provide a specially optimized stent design. Rather, this study focuses on the identification of factors that influence the mechanical behavior of L-PBF stents and on the development of a numerical methodology that could be used for the analysis and optimization of L-PBF stents.

### 2.2 Experimental framework

In the following, the respective experimental tests are described. A summary of the experimental tests is provided in Table 1.

**Table 1:** Overview of the experimental framework.

Method	Test specimens	Aim
**Mechanical testing**		
Tensile test	2 L-PBF flat specimens: 1 as-built 1 heat treated	Determination of the mechanical properties/flow curves of L-PBF 316L in the as-built and heat treated condition
Compression between two plates	10 L-PBF stents 4 as-built 4 heat treated 4 electropolished and heat treated	Determination of the global macroscopic response/radial force of the L-PBF stents under compression;
**Characterization**		
X-Ray computed tomography (X-ray CT)	3 L-PBF stents before and after compression: 1 as-built 1 heat treated 1 electropolished and heat treated	Reconstruction of L-PBF stent models for morphological and subsequent numerical analysis; Determination of L-PBF process-related geometrical irregularities
Scanning electron microscopy (SEM)	3 L-PBF stents: 1 as-built 1 heat treated 1 electropolished and heat treated	Determination of L-PBF process-related geometrical irregularities
Metallographic analysis	Metallographic sections of 2 L-PBF stents: 1 as-built 1 heat treated	Determination of the impact of heat treatment on the microstructure of L-PBF 316L stents

#### 2.2.1 Laser powder bed fusion process

The L-PBF stents and tensile specimens (DIN 50125 form E [30], thickness 1 mm) were made from gas-atomized 316L stainless steel powder with a particle diameter of 15 to 45 *μ*m (LPW, Rundkorn, United Kingdom). An SLM^®^ 250 machine (SLM Solutions Group AG, Lübeck, Germany) equipped with a 400 W Yb-fibre-laser was used with the following standard process parameters: laser power of 350 W, scanning velocity of 700 mm/s, focus position of 0, layer thickness of 30 *μ*m, hatch distance of 80 *μ*m, and rotation per layer of 83°. Furthermore, platform preheating of 100°C was employed. At maximum power (400 W) the laser has a spot diameter of 150 *μ*m. With the selected process parameters, a minimum strut diameter of about 200 *μ*m and a density ≧ 99% can be achieved, which is in a comparable range to the L-PBF CoCr stents previously published in Finazzi et al. [11]. The build direction was parallel to the longitudinal stent axis (Fig 1, blue arrow). The longitudinal struts at the bottom end of each stent were extended to serve as supports for the stents and to prevent the fusion of the stent spikes with the build platform. The stents were removed from the build platform by cutting these extensions. The tensile specimens were aligned in a standing position with the longitudinal (loading) axis of the specimen parallel to the build direction (BD) (sample orientation 0° to BD).

#### 2.2.2 Post-build treatments

Three different stent conditions were considered in this study: i) as-built, ii) heat treated, and iii) electropolished and heat treated condition. The stents in the as-built condition were only cut from the build platform and did not undergo any post-processing steps. The stents in the heat treated condition and the stents in the electropolished and heat treated condition are subjected to heat treatment at a temperature of 1050°C for 1 h and subsequently cooled inside the furnace. This heat treatment was intended to homogenize the microstructure, to decrease the yield strength and increase ductility, thus increasing the deformability of the stents and enabling stent crimping and expansion. The stents in the electropolished and heat treated condition were additionally electropolished after heat treatment using the electrolyte Poligrat E 268 at a temperature of 85°C with a voltage of 3.8 V and an electric current of 5.5-6 A for a duration of 4 minutes. Analogous to the stents, flat tensile specimens (DIN 50125 form E [30], thickness 1 mm) were prepared in the as-built and heat treated condition. The parameters of the heat treatment were analogous to the stents. To minimize the influence of surface roughness and thus to determine the bulk material properties, all tensile specimens are electropolished before testing (7 min, 85°C, 6 V, 5.5–6 A).

#### 2.2.3 Geometrical analysis

The morphology and internal defects of the L-PBF stents were determined by X-ray CT (Phoenix v|tome|xs 240-180, GE Sensing Inspection Technologies, Hürth, Germany) with a source voltage of 90 kV, an intensity of 390 *μ*A, and a voxel size of 8.5 x 8.5 x 8.5 *μ*m^3^. X-ray CT was performed for each stent in the undeformed condition as well as after compression tests. The X-ray CT data was further used for the reconstruction of the L-PBF stent models within the numerical analysis and their validation. For a more detailed analysis of the strut surfaces and local deformation concentrations, SEM (1450 VP, LEO Elektronenmikroskopie GmbH, Oberkochen, Germany) imaging was performed for each stent after the compression with parallel plates. The stent mass was determined using a monolithic weighing system Sartorius LA310S (Sartorius, Göttingen, Germany).

#### 2.2.4 Metallographic analysis

For the metallographic analysis of the influence of heat treatment on the microstructure of L-PBF 316L stents, metallographic sections of a stent were analysed for the as-built and heat treated conditions. To do so, the stents were cut lengthwise, embedded planarly in hot mounting resin, ground, mirror polished and etched in hot V2A etchant, and then analyzed with a light microscope following standard practice. The stents that were destructively characterized were manufactured together with the stents experimentally investigated here and subjected to the same heat treatment.

#### 2.2.5 Mechanical testing

To determine the material properties of L-PBF 316L, uniaxial tensile tests were performed on L-PBF 316L tensile specimens in the as-built and heat treated condition (S1 Fig). The tensile tests were performed at room temperature using a universal testing machine (Hegewald & Peschke Mess- und Prüftechnik GmbH, Nossen, Germany) with a constant loading speed of 1 mm/min and a loading cell of 50 kN. Strain was measured using an extensometer with an initial gauge length of 25 mm.

**Fig 2:**
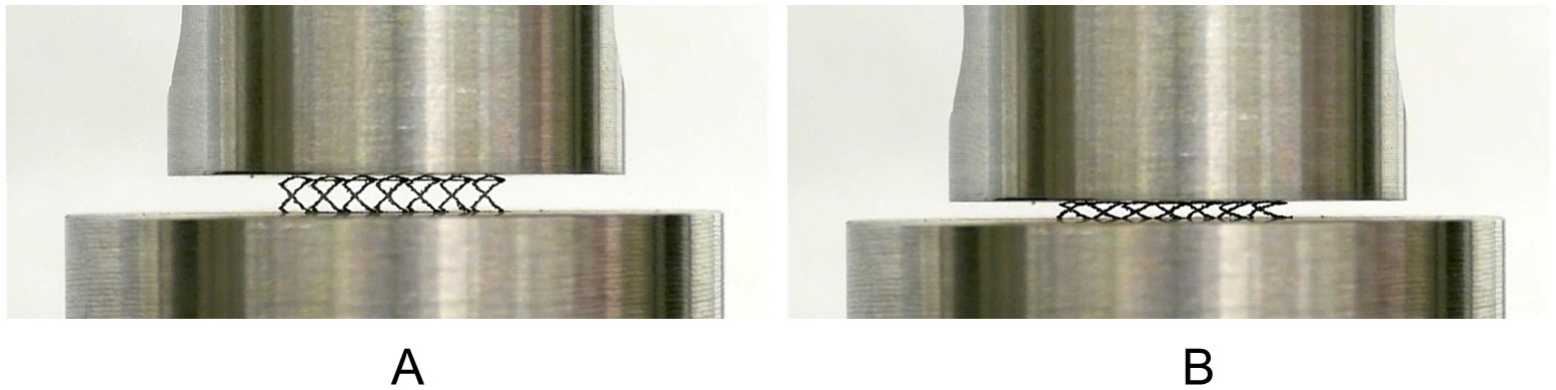
Experimental set up of the stent compression tests with parallel plates. A: Initial positioning of the stent between the two plates. B: Stent after compression between the two plates.

To assess the macroscopic mechanical behavior of the L-PBF stents under compression, room-temperature compression tests with parallel plates were performed (Fig 2). This test is commonly used to asses the crush resistance of stents following the standard ISO 25539-2 [29]. Therefore, a universal testing machine with a constant loading speed of 1 mm/min and a loading cell of 500 N was used. The stent was positioned between the two plates so that the longitudinal struts were aligned centrally and parallel to the plates. A total of twelve L-PBF stents were tested: i) four in the as-built condition, ii) four in the heat treated condition and iii) four in the electropolished and heat treated condition.

### 2.3 Computational framework

Within the computational framework, the finite element solver Abaqus/Explicit 2019 was used for the analysis of the L-PBF stents. Care has been taken to ensure that the ratio of kinetic to internal energy does not exceed a critical value of 5-10% during the major part of the process, so that the influence of inertial effects is negligible [31]. In addition, a mesh convergence study was carried out to select an adequate mesh density that would allow sufficient accuracy within a reasonable calculation time (S2 Fig).

#### 2.3.1 3D reconstruction of the selectively laser melted stents

**Fig 3:**
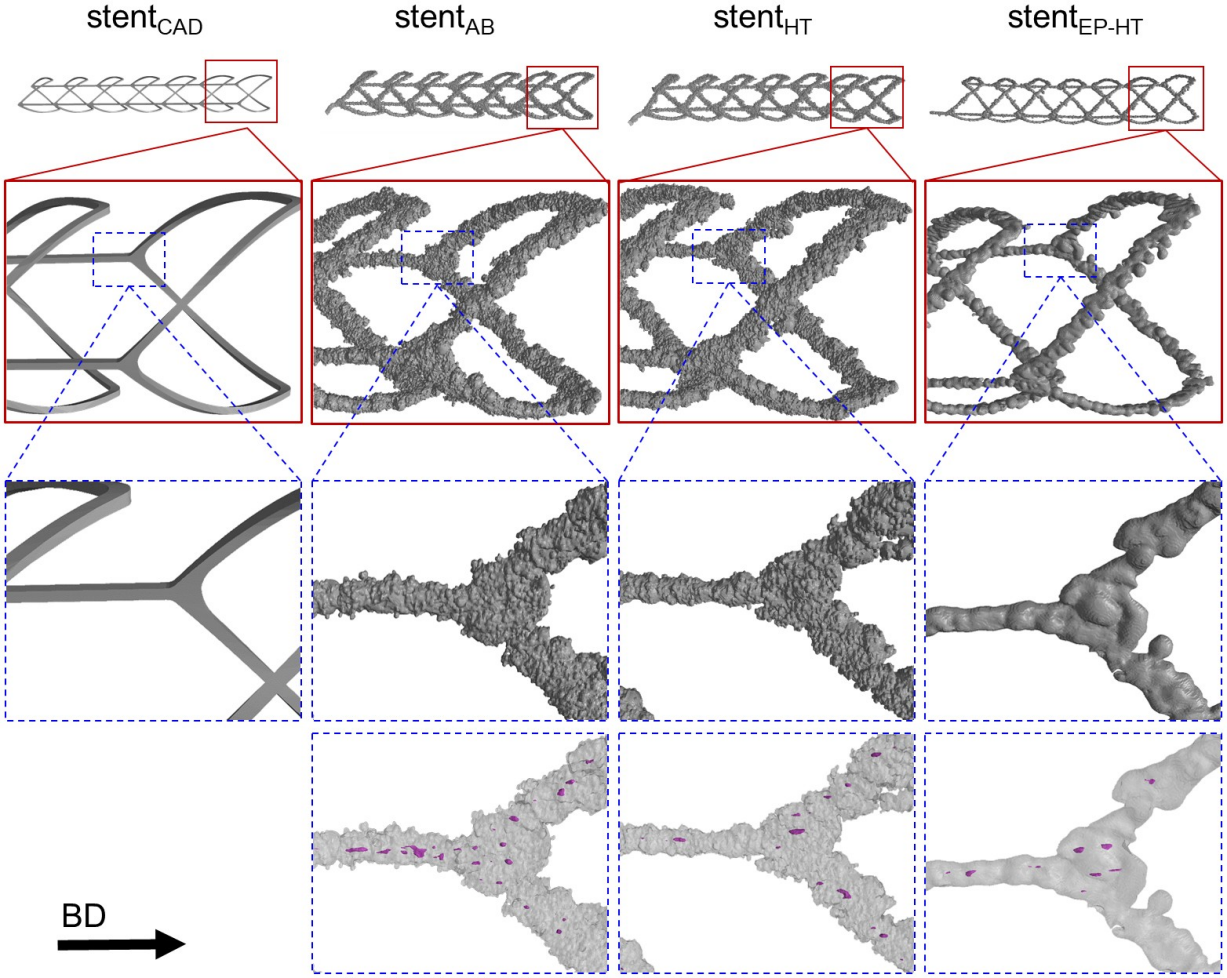
Stent configurations investigated in this study. (from left to right): idealized CAD model, stent_AB_ in the as-built (AB) condition, stent_HT_ in the heat treated (HT) condition, and stent_EP-HT_ in the electropolished and heat treated (EP-HT) condition. The laser powder bed fused stents are reconstructed based on X-ray computed tomography (CT) data. Pores within the struts are illustrated in magenta (bottom row). The build direction (BD) is indicated by an arrow.

Due to high numerical requirement, only one representative model for each investigated condition of the L-PBF stents was reconstructed from X-ray CT data and considered within this study: i) stent_AB_ representing the stents in the as-built condition, ii) stent_HT_ representing the stents in the heat treated condition and iii) stent_EP-HT_ representing the stent in the electropolished and heat treated condition. For the 3D reconstruction of the stents, the X-ray CT images were manually segmented based on their grayscale values using the threshold segmentation tool within the 3D segmentation software Simpleware ScanIP (Synopsis^®^, Mountain View, California, USA). Pores were explicitly considered as voids within the struts (Fig 3, magenta areas at bottom row). After the 3D reconstruction, the volume, surface area, porosity, and strut diameter were determined using the measurement tools within Simpleware ScanIP. The centerline of the stents was used to measure the strut diameter based on the best fit circle perpendicular to the centerline. Then, a finite element mesh of each 3D-reconstructed L-PBF stent was created in Simpleware ScanIP and subsequently imported into the finite element solver Abaqus/Explicit 2019. Besides the reconstructed stent models, the idealized CAD model stent_CAD_ on which the L-PBF stents are based, was further considered in this study which allows for the comparison between the actual and as-designed mechanical response of L-PBF stents. The basic morphology of the investigated L-PBF stents is given in Fig 3.

**Fig 4:**
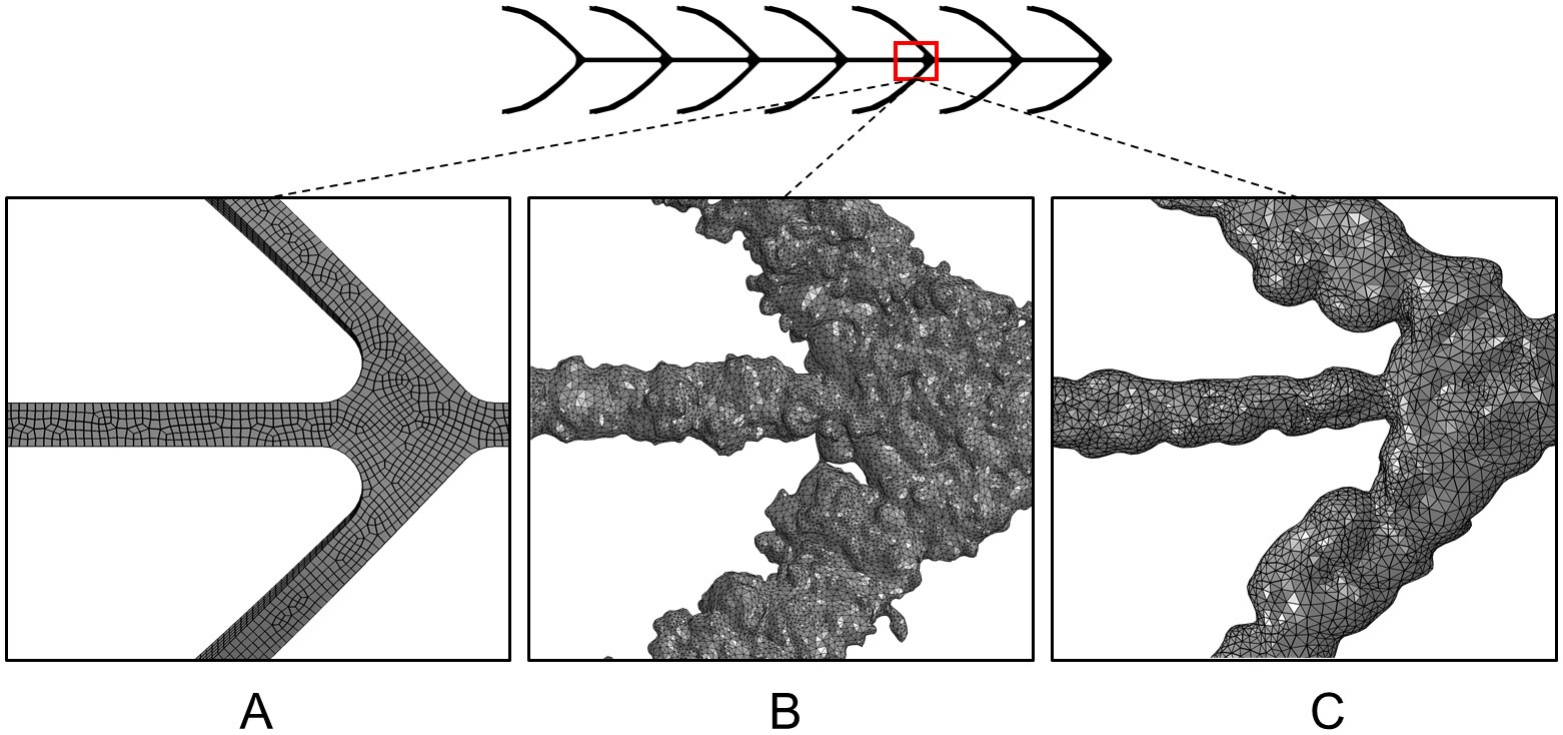
Illustration of the discretization of the stent models. A: Magnified view of mesh for the idealized CAD model stent_CAD_. B: Magnified view of mesh for stent_AB_ in the as-built condition (also representing stent_HT_ due to similar morphology). C: Magnified view of mesh for stent_EP-HT_ in the electropolished and heat treated condition.

Due to the high geometric complexity, the three L-PBF stents were modeled with linear tetrahedral elements with an element size of 0.02 mm (Abaqus element type C3D4). The as-designed CAD model stent_CAD_ of the L-PBF stents was meshed with eight node linear brick elements with reduced integration and hourglass control with an element size of 0.02 mm Abaqus element type C3D8R). An overview of the investigated stent models and their discretization are given in Table 2, and the investigated meshes are exemplary highlighted in Fig 4.

**Table 2:** Investigated stent models and their discretization.

Model	Stent configuration description	element type	number of elements
stent_CAD_	Intended stent design (CAD model)	C3D8R	175 790
stent_AB_	Reconstructed L-PBF stent in the as-built condition	C3D4	4 248 831
stent_HT_	Reconstructed L-PBF stent in the heat treated condition	C3D4	2 867 569
stent_EP-HT_	Reconstructed L-PBF stent in the electropolished and heat treated condition	C3D4	1 284 450

#### 2.3.2 Material description

**Fig 5:**
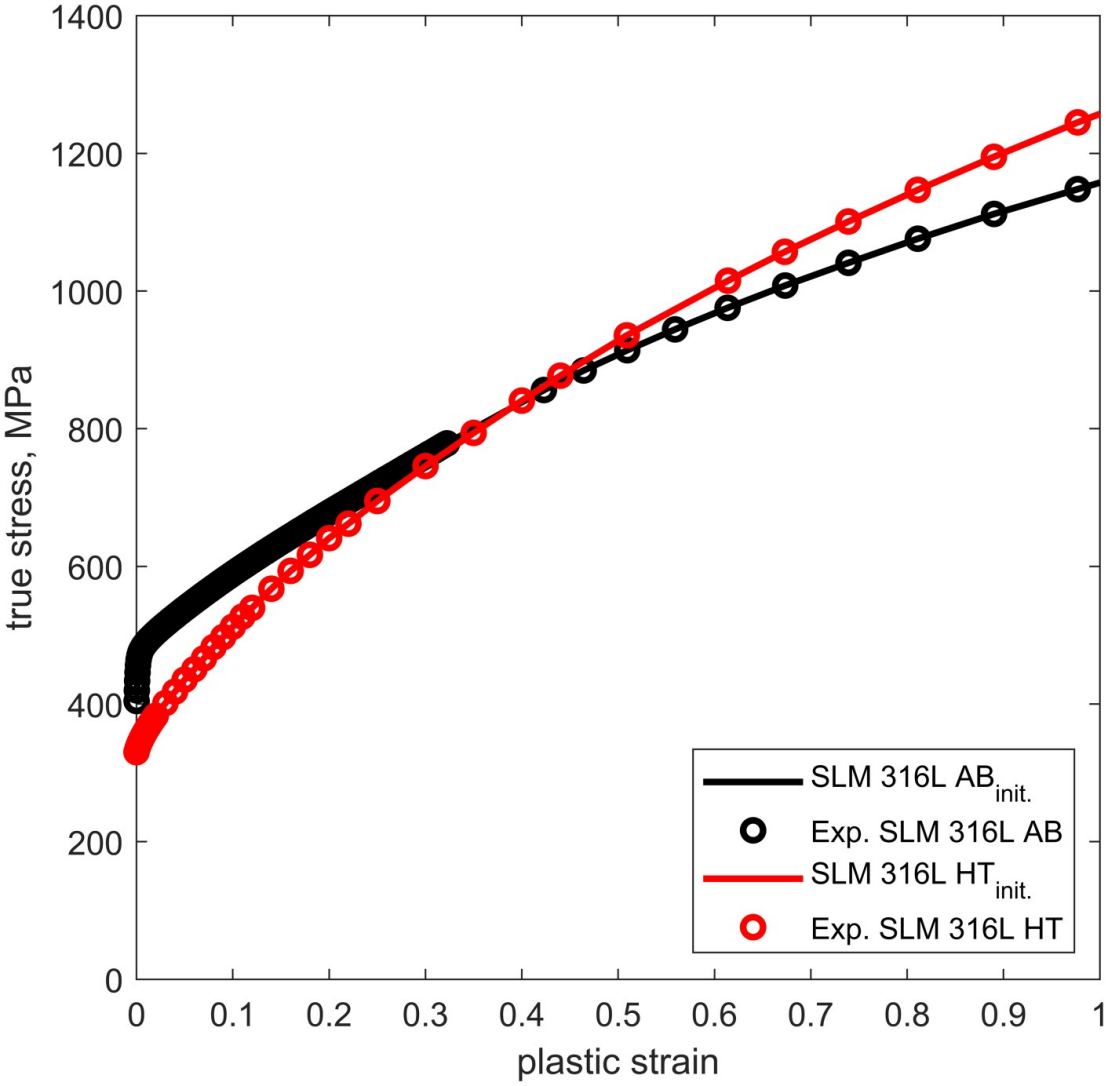
Flow curves derived from the results of the uniaxial tensile test for laser powder bed fused flat specimens. The tensile specimens have a thickness of 1 mm. The flow curve representing the as-built condition is indicated by the black line and the flow curve representing the heat treated condition by the red line. The experimental data points are marked by spheres.

The as-built and heat treated material behavior of the stents is described by a von Mises plasticity model with isotropic hardening behavior. The elastic properties were approximated by a Young’s modulus of 193 GPa and a Poisson’s ratio of 0.3 [32]. The plastic material properties were determined from the uniaxial tensile test by transferring the experimentally determined engineering stress-strain curves into true values. The flow curves were extracted until uniform elongation. Thereupon, strain hardening was considered by Hollomon’s power law ($\bar{\sigma }\left({\bar{\epsilon }}^{pl}\right)=A\cdot {\left({\bar{\epsilon }}^{pl}\right)}^{n}$) (Fig 5). Corresponding Hollomon power law parameters for the as-built condition are A_*AB*_ = 1157 MPa and n_*AB*_ = 0.35 and for the heat treated condition are A_*HT*_ = 1257 MPa and n_*HT*_ = 0.44. The flow curves were implemented in tabular form in Abaqus/Explicit and assigned to the respective stent models.

#### 2.3.3 Stent compression simulation

**Fig 6:**
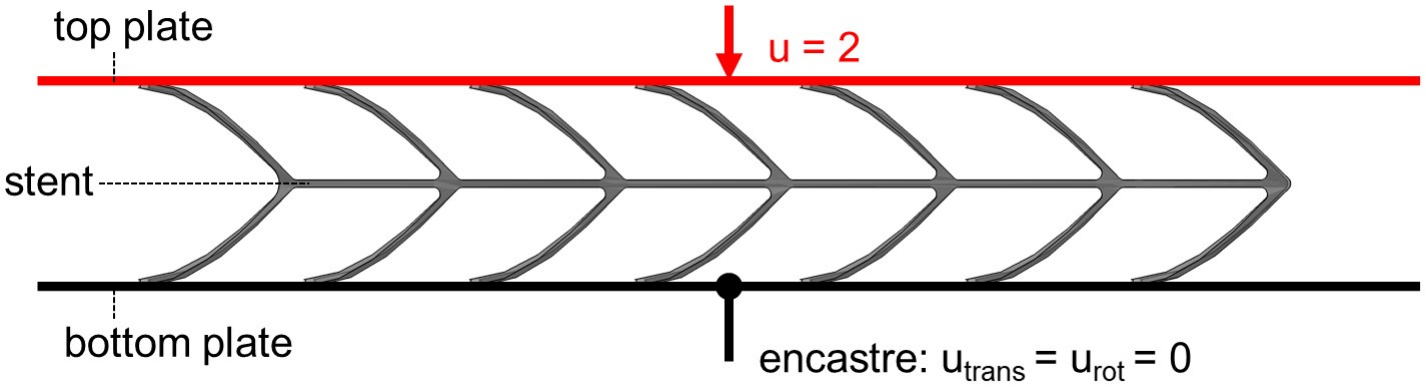
Illustration of the set-up of the stent compression simulations.

The top and bottom plate of the compression test were implemented as analytical rigid surfaces. The reconstructed stents were positioned analogously to the experimental setup, with the longitudinal struts being aligned centrally and parallel to the plates. Stent compression was initiated by applying a linearly increasing displacement of 2 mm in the negative vertical direction to the upper plate. The bottom plate was encastred by constraining all translational and rotational degrees of freedom (*u*_*trans*_ = *u*_*rot*_ = 0). Contact between the plates and the stent, as well as stent self-contact, were invoked using the general contact algorithm (friction-less, penalty method) within Abaqus/Explicit. The simulation set-up is illustrated in Fig 6.

An overview of the L-PBF stent compression simulations (virtual test matrix) is given in Table 3. Each stent model was assigned the material properties that correspond to its respective material state (as-built, heat treated). For the stent_CAD_, both material states were considered to provide a reference for comparing the intended and the actual L-PBF stent behavior.

**Table 3:** Overview of the models used in the stent simulations.

Model	Stent configuration description	Material condition	Porosity
stent_*CAD*, *AB*_	Idealized CAD stent	as-built	no
stent_*CAD*, *HT*_	Idealized CAD stent	heat treated	no
stent_AB_	Reconstructed L-PBF stent in the as-built condition stent	as-built	yes
stent_HT_	Reconstructed L-PBF in the heat treated condition	heat treated	yes
stent_EP-HT_	Reconstructed L-PBF in the electropolished and heat treated condition	heat treated	yes

#### 2.3.4 Stent crimping and expansion simulations

A previously published FEA of stent expansion using a balloon catheter was used for this analysis. For a detailed description of the simulation approach please refer to Ref. [33]. In addition to the actual analysis of the expansion behavior, the applicability of methods/models already established in the field of conventional laser-cut stents to L-PBF stents can be investigated. Stent_AB_ and stent_HT_ represent L-PBF stent morphologies without surface treatment and thus present a similar morphology and geometrically induced stiffness. Stent_EP-HT_ represents the stent morphology after surface treatment (electropolishing), resulting in a smoothing of the strut surfaces, a reduction of the strut diameter and thus a reduction of the geometrically induced stiffness. It is, therefore, expected to have a different expansion behavior compared to the stents without surface treatment. Again, the expansion simulations of the CAD stent (stent_CAD_) were performed for both material conditions (as-built, heat treated) to determine the differences between the actual and as-designed expansion behavior. Since the investigated stent models were identical to those used in the stent compression simulation, it is referred to Table 3 (virtual test matrix) for an overview of the stent crimping and expansion simulation.

## 3 Results and discussion

In this section the effects of L-PBF and post-processing steps on stent morphology and mechanical behavior of L-PBF stents under compression are presented and discussed. Subsequently, the influence of the size effect on the mechanical behavior of L-PBF 316L is derived and discussed by comparing the numerically predicted compression behavior of the stents using bulk material properties (derived from the tensile specimens) with the experimentally determined data. Thereupon the results of the numerical analysis of stent compression and expansion of the reconstructed L-PBF stents are presented and compared to those of the intended CAD stent models. Finally, the limitations and further aspects of the work are discussed.

### 3.1 Morphological analysis

The basic morphology of the L-PBF stent configurations has already been shown in Fig 3. The L-PBF stents show the typical process-related geometric irregularities similar to L-PBF lattice structures, e.g. surface roughness, deviation in the strut cross-sectional shape and the strut diameter, strut waviness, tapers, and porosity [16–20, 34–36]. These geometric irregularities are attributed to the layer-by-layer manufacturing, local energy input and melting of the powder, different heat dissipation properties of the powder and the solidified material, as well as L-PBF process parameters [1, 34, 35, 37–39]. In the following, the individual irregularities are described in detail and their causes discussed.

#### 3.1.1 Strut cross-sectional shape

**Fig 7:**
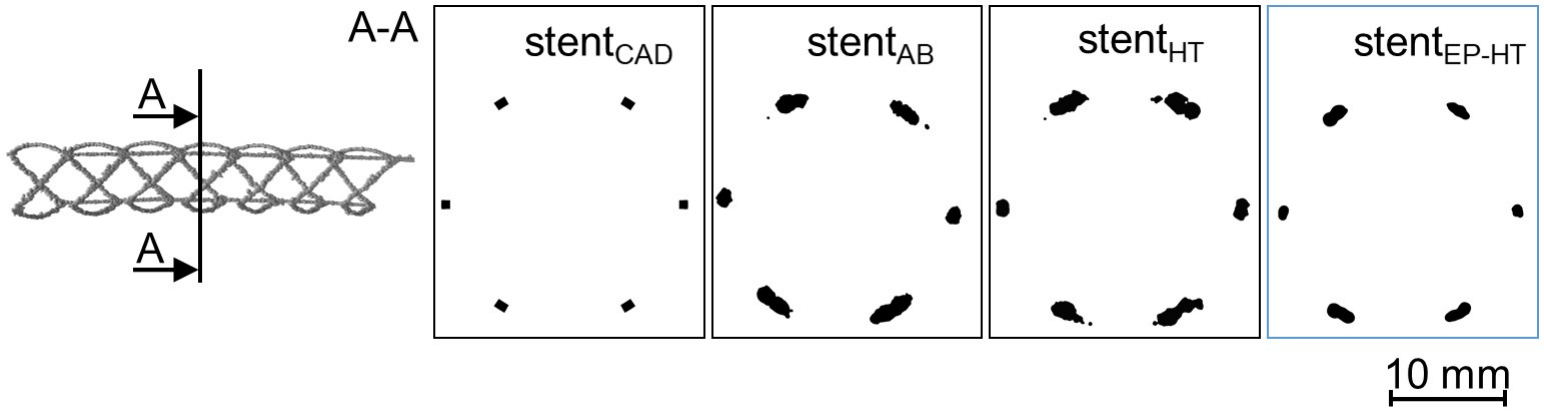
Deviation in strut shape between the as-designed and actual laser powder bed fused (L-PBF) stents. Stent_CAD_ correspond to the morphology of the as-designed stent CAD model. Stent_AB_, stent_HT_, and stent_EP-HT_ correspond to L-PBF stents in the as-built (AB), heat treated (HT), and electropolished and heat treated (EP-HT) conditions, respectively.

The L-PBF struts have a circular cross section instead of exhibiting the intended quadratic cross-sectional shape of the stent CAD model (Fig 7). This observation is in accordance to the findings of Finazzi et al. [11] on L-PBF CoCr stents. Due to the small structure size, the stent struts are only exposed in one hatch. The circular cross-sectional shape can thus be attributed to the laser spot and the melt pool geometry. Due to the high surface roughness, the contour of the cross-section of the stents without surface treatment (stent_AB_, stent_HT_) further appears non-uniform in contrast to the electropolished stent (stent_EP-HT_).

#### 3.1.2 Surface roughness and strut waviness

**Fig 8:**
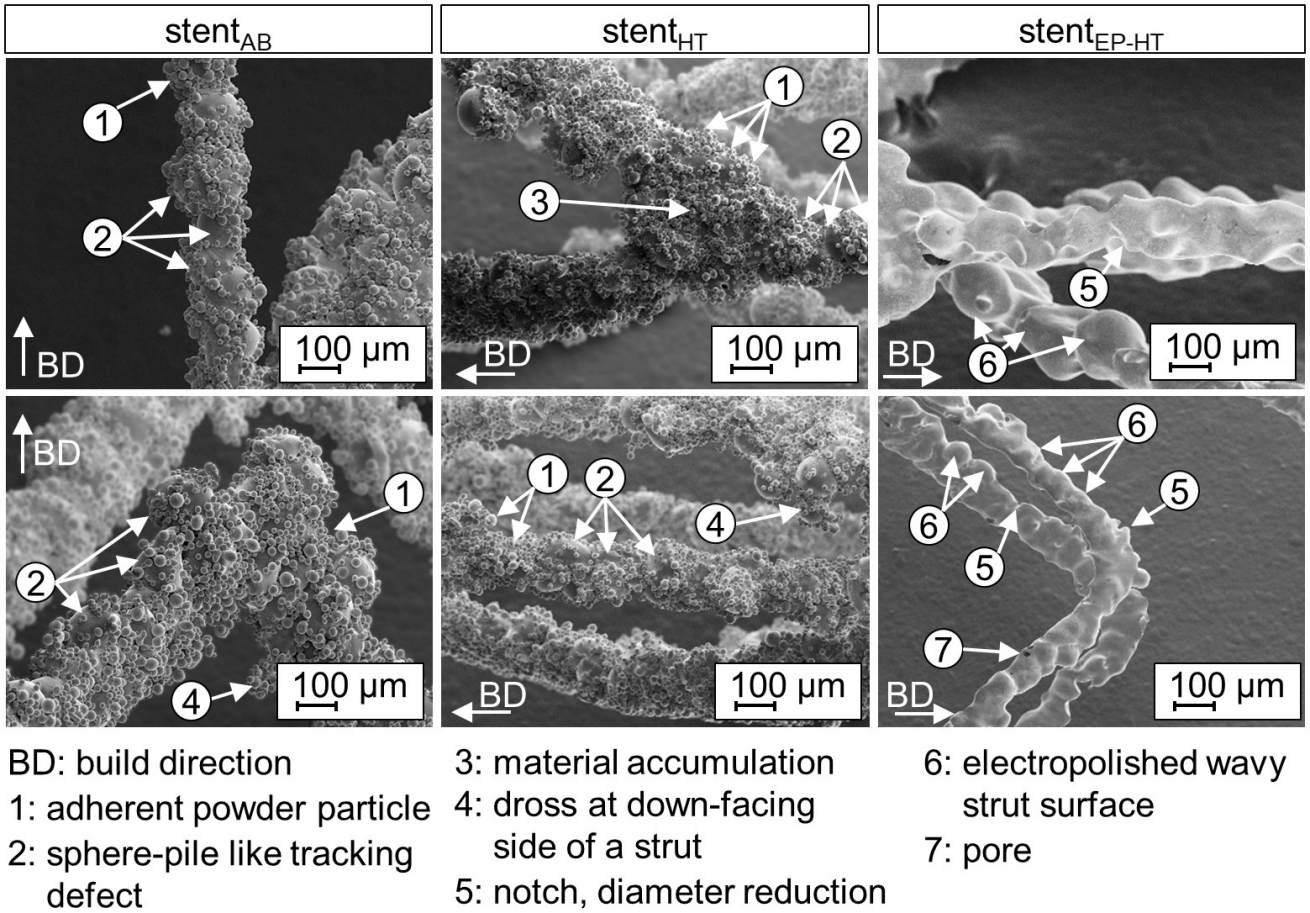
Scanning electron microscopy images highlighting the geometric irregularities related to the laser powder bed fusion process. Stent_AB_, stent_HT_ and stent_EP-HT_ correspond to laser powder bed fused stents in the as-built (AB), heat treated (HT), and electropolished and heat treated (EP-HT) conditions, respectively.

L-PBF stents without surface treatment (stent_AB_, stent_HT_) have high surface roughness caused by partially melted and adhering powder particles on the strut surface, regardless of the material condition (as-built, heat treated) (Fig 8: ①②). Surface roughness and strut waviness are mainly attributed to stacked solidified melt pools (sphere-pile-like defects) and adhering or partially melted powder particles [1, 34, 38]. These sphere-pile-like defects result from the layer-by-layer manufacturing process (staircase effect) and from discontinuous melting tracks due to insufficient control of the exact position of the melt pool at such small strut diameters [35]. The adhesion of powder particles is attributed to thermal diffusion caused by the high temperature gradient between the powder material and the solidified struts [1]. At the strut edge, the powder is occasionally not completely melted, resulting in partially melted powder particles adhering to the strut surface [1, 38].

Material ablation during electropolishing results in the removal of these adherent powder particles and the compensation/rounding of local protrusions without extensively alternating the cross-sectional shape. However, strut waviness and significant deviation in cross-section cannot be fully compensated for via electropolishing (Figs 7 and 8: ⑤⑥).

#### 3.1.3 Strut thickness and dimensional error

The strut thickness of the L-PBF stents is overall increased and irregular over the strut length; although, local reduced diameters/tapers and notches occur (Figs 3 and 8: ⑤). For the struts inclined relative to build direction, even larger strut diameters and an increased number of adhering powder particles are observed (Table 4, Fig 8). In relation to the as-designed stent_CAD_, the strut diameter is increased by about 2 and 2.6 times for the longitudinal struts and inclined struts, respectively, of the L-PBF stents without surface treatment (stent_AB_, stent_HT_) and by about 1.3 and 2 times for the longitudinal struts and inclined struts, respectively, of the electropolished stent_EP-HT_. Further, material accumulations occur at the nodes between inclined and longitudinal connecting struts (Fig 8: ③).

**Table 4:** Analysis of the basic morphological parameters of the laser powder bed fused stents.

Stent	D_*stent*_, mm	D_*strut*, *longitudinal*_, *μ*m	D_*strut*, *inclined*_, *μ*m	surface area, mm^2^	mass, g	porosity, %
stent_CAD_	3.00	quadratic 100 x 100		46.54	0.0096	—
stent_AB_	3.22	200 ± 25	250 ± 60	148.68	0.0412	0.17
stent_HT_	3.20	210 ± 35	260 ± 110	153.86	0.0421	0.18
stent_EP-HT_	3.20	130 ± 30	200 ± 70	89.03	0.0223	0.20

Stent_AB_, stent_HT_ and stent_EP-HT_ correspond to the actual laser powder bed fused stents configurations determined from their 3D reconstruction from X-ray CT in the as-built (AB), heat treated (HT), and electropolished and heat treated (EP-HT) conditions, respectively. Stent_CAD_ corresponds to the as-designed stent CAD model.

The increased strut diameter can be attributed to the fact that the melt pool has a larger diameter than the original laser spot [35, 40]. For struts that are inclined relative to the build direction, this effect is exacerbated by the staircase effect and the overmelting of the downward facing strut side [34, 37]. The overmelting of the downward facing side is due to lower thermal conductivity of the powder compared to the bulk material. The heat is therefore preferably dissipated via the solidified strut. As inclined struts are partially built on powder, heat accumulation occurs on the downward facing side, which leads to overmelting, the formation of dross and spatter, and the adhesion of powder particles [34, 37] (Fig 8).

All of these irregularities result in an overall increase of the L-PBF stent mass and surface area compared to the as-designed stents. The stents without surface treatment (stent_AB_, stent_HT_), thus, have about 4.3/4.4 times, and the electropolished stent_EP-HT_ about 2.4 times, the mass of the as-designed stent_CAD_ (Table 4). The mass of the as-designed stent_*CAD*_ was determined in Abaqus under the assumption that the stent has no internal porosity and a material density of *ρ* = 9700 kg/m^3^.

#### 3.1.4 Porosity

The average internal porosity of the L-PBF stents determined from X-ray CT data, is about 0.17 to 0.2% thus implying high densification (≧ 99%) (Table 4). The average pore size was 31 *μ*m with a minimum pore size of 8.5 *μ*m. The minimum pore size corresponds to the minimum resolution of the CT (voxel size 8.5 *μ*m) thus neglecting the presence of smaller pores, e.g. gas pores. Porosity is a well-known characteristic of AM commonly related to entrapped gas, lack of fusion, unmelted or partially melted particles and delamination between the layers [41, 42]. Although the determined porosity is low, pores must be considered critical due to their strength-reducing effect and their negative impact on fatigue strength, especially since the pore size is of the same order of magnitude as the stent struts [19, 43]. The occurrence of pores is almost unavoidable in L-PBF. To minimize porosity, further comprehensive studies are required that investigate the effect of machine-to-machine variability, powder characteristics, and process parameters.

#### 3.1.5 Further considerations: Process parameters and stent design development

Experimental studies on L-PBF lattice structures have shown that the extent of the morphological irregularities depends strongly on the process parameters used, such as laser power and scanning speed [39]. Increased laser power, in combination with a reduced scanning speed, is related to larger melt pool diameters due to increased overmelting. This, in turn, leads to an increase in strut diameter and an intensification of sphere/plate-pile defects. A reduced laser power in combination with a reduced scanning speed, on the other hand, is related to turbulent melt pool behavior and thus increased strut irregularities and waviness. In summary, L-PBF causes morphological deviations between the as-designed and actual structure, which have a very strong impact on the mechanical properties, especially for small structures, including the L-PBF stents investigated in this work. A component design based exclusively on the CAD models without taking the morphological irregularities into account could, therefore, lead to unintended mechanical response of the final L-PBF component, which is further discussed below.

### 3.2 Mechanical behavior of laser powder bed fused stents under compression

**Fig 9:**
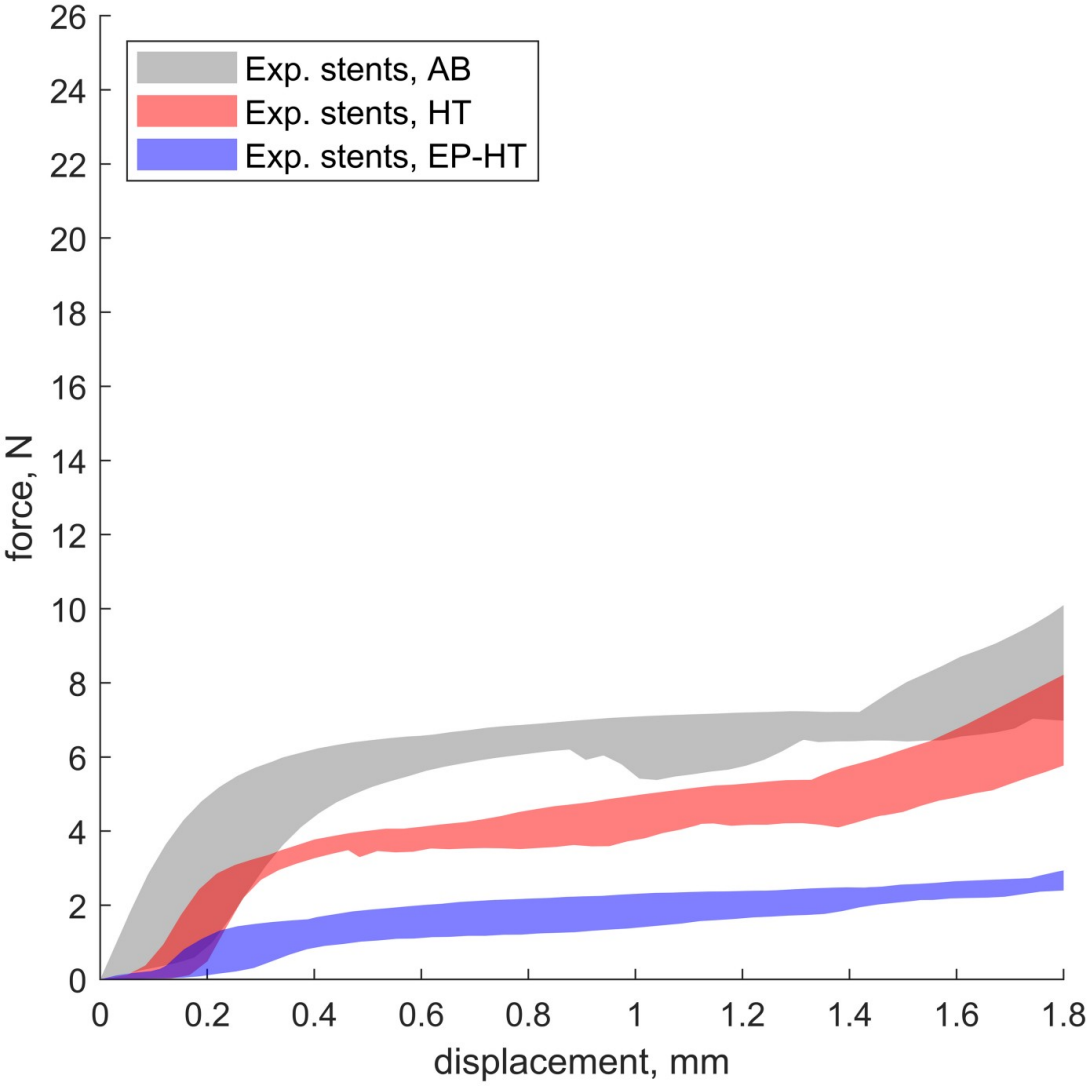
Experimental determined mechanical response of laser powder bed fused stents under compression. The shaded curve areas show the experimental results of the compression tests of the respective stent configuration (as-built stents (AB, gray area), heat treated stents (HT, light red area), electropolished and heat treated stents (EP-HT, light blue area). To indirectly consider the impact of the geometrically induced stiffness, the mean mass $\bar{m}$ of the respective stent configuration is provided in the legend.

The macroscopic mechanical behavior of the experimentally tested L-PBF stents under compression (Fig 9) corresponds to the characteristic deformation behavior of metal foams [44], with an initial linear-elastic region, followed by high plastic deformation characterized by a plateau with only a small gradient and subsequent densification characterized by a high gradient. The L-PBF stents in the as-built condition (Fig 9: gray area) have the highest compressive strength, followed by the heat treated stents (Fig 9: light red area) and the electropolished and heat treated stents stent_EP-HT_ (Fig 9: light blue area). Geometric irregularities have been found to cause an increase in the strut diameter *D*_*strut*_ and thus an increase in the section modulus *S*_*strut*_ of the struts ($S_{strut}=\pi D_{strut}^{3}/32\;\approx \;0.1D_{strut}^{3}$) and the total stent mass. Because a detailed measurement of the strut diameters was only performed on the reconstructed stents, the average mass $\bar{m}$ of the respective stent configuration is provided in the legend of Fig 9 and in Table 5 for comparison purposes. The strut thickness is thus deduced based on the constant topology and the comparable mass of the respective stent configuration. The radial force at 50% compression F_rad.50%_ (i.e. a uniaxial displacement of 1.5 mm for an initial intended outer stent diameter of 3 mm) which is commonly used for the assessment of the radial strength of stents [29], is presented in Table 5. With radial forces F_rad.50%_ of 2.39 ± 0.23 N, the electropolished and heat treated stents exhibit similar values as reported for conventional stents such as the PROMUS Element^*TM*^ (F_rad.50%_ = 2.89 ± 0.28 N) or the Xience Prime (F_rad.50%_ = 2.73 ± 0.24 N) [45].

**Table 5:** Experimentally determined radial force at 50% compression F_rad.50%_ and the corresponding mass *m* of the respective stent configuration.

Stent configuration	F_rad.50%_, N	m, g
as-built	7.11 ± 0.63	0.0423 ± 0.0009
heat treated	5.87 ± 0.49	0.0421 ± 0.0005
electropolished and heat treated	2.39 ± 0.23	0.0245 ± 0.0015

As the investigated stents exhibit basically the same topology, the observed difference in radial strength are attributed to the different geometrically induced stiffness and the material condition of the respective L-PBF stent configuration.

The stents in the heat treated condition (Fig 9: light red area) and the stents in the electropolished and heat treated state (Fig 9: light blue area) feature the same material condition. The L-PBF stents are composed of solidified polycrystalline material. Therefore, material removal by electropolishing does not alter the material related stiffness of the stent, since the strength of the removed material is equal to that of the remaining stent material. The increased stiffness of the heat treated stents without surface treatment is therefore mainly due to their increased geometrically induced stiffness resulting from the increased strut diameter due to geometric irregularities discussed above. Electropolishing results in a smoothing of the strut surface by removing or reducing adhering and partially melted powder particles, as well as larger protruding surface features. Therefore, the strut diameter and thus the geometrically induced stiffness are reduced and the deformability of the stent is increased. During stent compression, deformation occurs predominantly in the inclined struts. With an average diameter D_strut,inclined_ of 200 *μ*m, the inclined electropolished struts exhibit only 45% of the section modulus S_strut, EP-HT_ of the stent struts without surface treatment (D_strut,inclined_ = 260 *μ*m) which causes the reduction of the radial force F_rad.50%_ of the electropolished stents by approximately 41% compared to the solely heat treated stents.

Due to their similar morphology, the stents in the as-built (stent_AB_) and the stents in the heat treated condition (stent_HT_) have similar strut diameters and thus similar geometric induced stiffness. The reduced radial strength of the heat treated stents (Fig 9, light red area) is therefore attributable to alterations in material properties following heat treatment. The applied heat treatment was aimed at reducing the yield strength and increasing the ductility to enable stent crimping and expansion. In the as-built condition, material comprising the L-PBF 316L stent shows almost ideal elastic-plastic behavior. In the heat treated condition, reduced yield strength and increased hardening behavior are present (S1 Fig). These observations are consistent with findings in the literature and are therefore only briefly discussed [26, 46].

**Fig 10:**
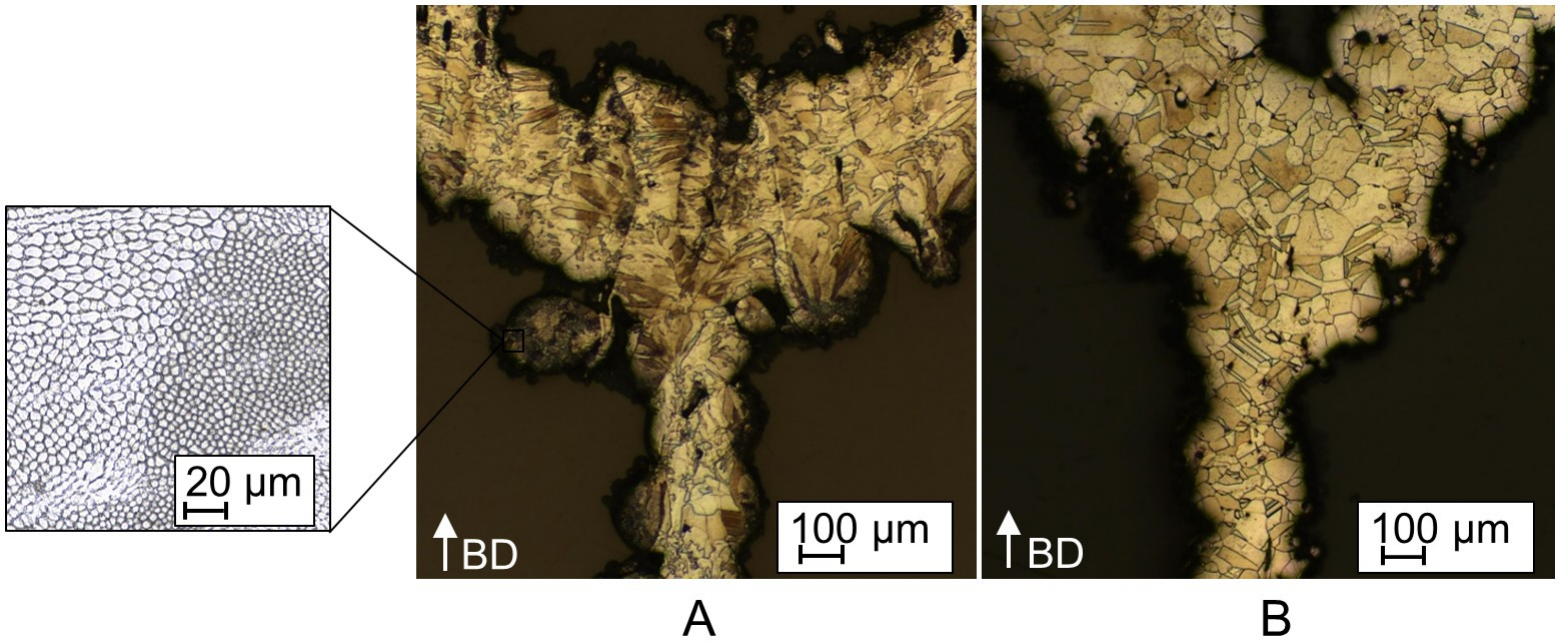
Metallographic sections of a laser powder bed fused (L-PBF) stents. A: Metallographic section of a L-PBF stent in the as-built condition highlighting the observed substructure. B: Metallographic section of a L-PBF stent in the heat treated condition.

The different mechanical properties between as-built and heat treated condition are attributed to differences in the microstructure and to the relief of residual stresses after heat treatment [26, 47, 48]. In the as-built condition, a microstructure typical for AM is observed, consisting of overlapping melt pools, coarse elongated grains, and the presence of a substructure (Fig 10A). The substructure is classified in literature as *δ*-ferrite, a strength-enhancing second phase [49]. Due to the high temperature gradients and densification rate, high dislocation densities and residual stresses occur in the as-built condition of L-PBF materials causing the material to undergo saturation with respect to strain hardening [28, 50, 51]. During heat treatment, the dislocation density is decreased, the residual stress are relieved and the dislocations act as nucleation points for recrystallization [26, 28, 47, 52]. The recrystallized microstructure is characterized by rather equiaxed coarse grains (Fig 10B), causing the reduction in yield strength (S1 Fig) [26]. Besides recrystallization, the heat treatment of L-PBF 316L is associated with the reduction/dissolution of the *δ*-ferrite phase as a strength-enhancing second phase, which explains the reduced yield strength [26].

### 3.3 Numerical analysis of the compressive behavior of L-PBF stents

#### 3.3.1 Impact of size effect on the mechanical behavior of laser powder bed fused stents

**Fig 11:**
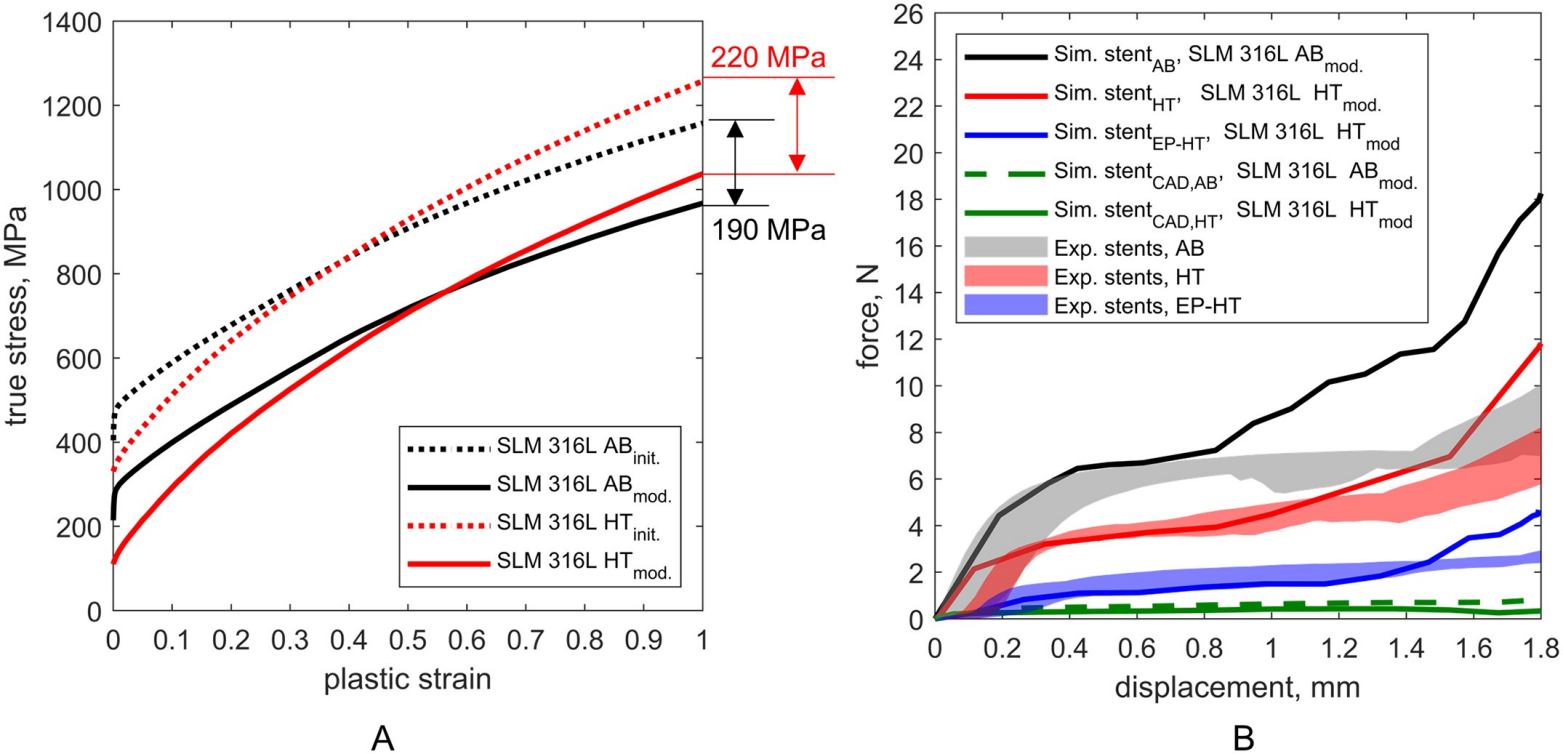
Mechanical response of laser powder bed fused (L-PBF) stents. A: Modification of the initial flow curves which were derived from flat tensile test to allow for the description of the L-PBF 316L stent material in the as-built and heat treated condition. B: Comparison of the experimental determined global mechanical response of the stents under compression (shaded curve areas) with the numerical predicted response of the respective reconstructed stent model configuration (solid lines) using the modified flow curves (L-PBF 316L AB_mod._, HT_mod._). Stent_AB_, stent_HT_ and stent_EP-HT_ correspond to the reconstructed stents in the as-built (AB), heat treated (HT), and electropolished and HT (EP-HT) conditions and, respectively. Stent_CAD,AB_ and stent_CAD,HT_ correspond to the as-designed stent CAD models with the as-built and heat treated material properties, respectively.

The numerical analysis of stent compression was initially investigated using the L-PBF 316L flow curves determined from flat specimens (Figs 5 and 11A: L-PBF 316L AB_*init*_, HT_*init*_). The predicted force-displacement curves of the L-PBF stent compression simulations using theses flow curves showed large deviations from the experimentally determined curves (S3 Fig). These deviations can be attributed to the size effect that has also been observed for L-PBF lattice structures [19, 21, 22]. The size effect is caused by the differences in microstructure due to different thermal histories of small and large L-PBF structures, as well as large surface-to-volume-ratio, the increasing defect density, and surface roughness of smaller structures [21, 22]. The determination of the exact origin of the strength-reducing mechanisms was not investigated in this study. However, it has been shown that the size effect has a strong influence on L-PBF 316L and has to be considered within the development of L-PBF stents.

Due to the size effect, the mechanical properties of L-PBF stents (strut thickness 0.1 mm) cannot be approximated by the flow curves derived from tensile flat samples (thickness 1 mm). It is therefore necessary to adjust the properties of the L-PBF 316L stent material to meet the experimentally determined global stent response. In the literature, tensile test data are available for L-PBF 316L micro-specimens in the as-built condition with diameters comparable to the stent struts [53–55], but not for specimens after heat treatment. Thus, for reasons of consistency, the flow curves of the as-built and heat treated L-PBF 316L were adjusted within the FEA of stent compression until the numerically predicted and experimentally determined force-displacement curves for stent_AB_ and stent_HT_ match. To accomplish this, the yield strength was systematically reduced (Fig 11A: L-PBF 316L AB_mod._, HT_mod._), assuming that the size effect has no influence on the hardening behavior of L-PBF 316L as been shown in Ref. [19]. Yield strengths *σ*_y_ of 271 MPa and 117 MP are determined to describe the L-PBF 316L stent material in the as-built and heat treated conditions, respectively. This corresponds to a reduction of yield strength *σ*_y_ by 41% in the as-built and by 59% in the heat treated condition compared to the initial material properties derived from the tensile tests.

**Fig 12:**
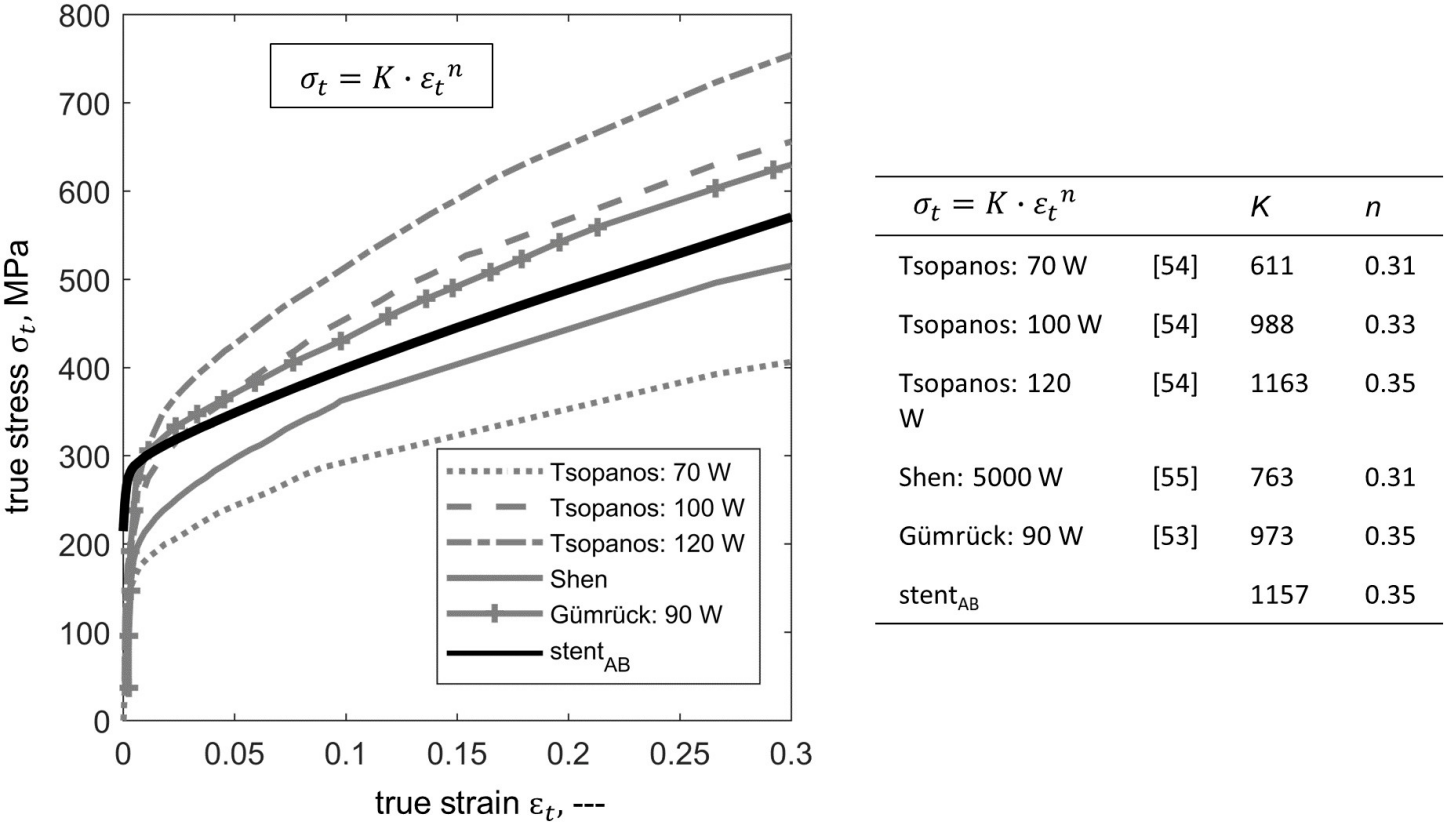
Comparison of the determined flow curve of the laser powder bed fused (L-PBF) 316L stent material with the flow curves and corresponding hardening parameters obtained from experimental data of L-PBF 316L micro-specimens from the literature [53–55].

To justify this modification, the determined L-PBF 316L flow curve in the as-built condition was compared with the flow curves obtained from experimental data of L-PBF 316L micro-specimens from the literature [53–55] (Fig 12). The comparable strain hardening parameters of the flow curves justify the modification of the flow curve in the as-built condition. The observed strength reduction is further consistent with the findings of Karamooz Ravari et al. [19] based on the mechanical behavior of single lattice struts with a diameter of 200 *μ*m. They found a reduction in the yield strength *σ*_y_ of the single struts of about 25% compared to the base material. The further decrease in strength observed in this study can be attributed to the large surface-to-volume ratio and the resulting high affinity for strength reducing effects due to surface irregularities.

Because no literature data on heat treated micro-specimens were available, the flow curve of the L-PBF 316L stent material in the heat treated condition was verified by means of the compression simulation of the electropolished and heat treated stent. Therefore, after fitting material properties based on the mechanical response of stent_HT_ (heat treated condition), the same material properties are then applied in the simulation of stent_EP-HT_ (electropolished and heat treated condition) to provide a validation of the adjusted material properties of the heat treated L-PBF stents. A good agreement with the experimentally determined force-displacement curves is achieved, thus verifying the adjusted flow curve (Fig 11). The subsequent simulations were all performed using the modified flow curves.

As shown above, after adjusting the material properties, the macroscopic mechanical behavior of L-PBF stents under compression can be predicted by FEA (Fig 11B). Within the FEA of the L-PBF stents, large differences between the actual (reconstructed) and the as-designed stent model were observed. As apparent from Fig 11B, the numerical prediction based on the stent_CAD_ leads to a significant underestimation of the compression strength compared to the reconstructed stent models, i.e. by a factor of 11 for the stents without surface treatment (stent_AB_, stent_HT_) and 4 for the stent with surface treatment (stent_EP-HT_). The increased stiffness of the actual L-PBF stents can be attributed to the increased geometrically induced stiffness of the stent struts resulting from the geometric irregularities and thus the increased strut diameter discussed above. It is further likely that the increased compression strength of the actual L-PBF stents depends not only on the overall global increase in mass, but also on the local mass distribution. In the L-PBF stent, material accumulations at the nodes (connecting points of the struts) are observed (Fig 8). In these regions, the largest deformations generally occur during compression. Thus, the local increase in stiffness due to material accumulation at the nodes could contribute to the global increase in stiffness.

The absence of a force increase in the posterior part of the force-displacement curve also indicates that the stent_CAD_, unlike the reconstructed stents, is not densified during the compression. With regard to the influence of the heat treatment, the compressive strength of the CAD stent model in the as-built condition (stent_CAD,AB_, green dashed line) is increased when compared to the heat treated condition (stent_CAD,HT_, green solid line). Again, given the relationship between the flow curves for the as-built and heat treated material condition (Fig 11A), this trend is not surprising.

#### 3.3.2 Qualitative validation

**Fig 13:**
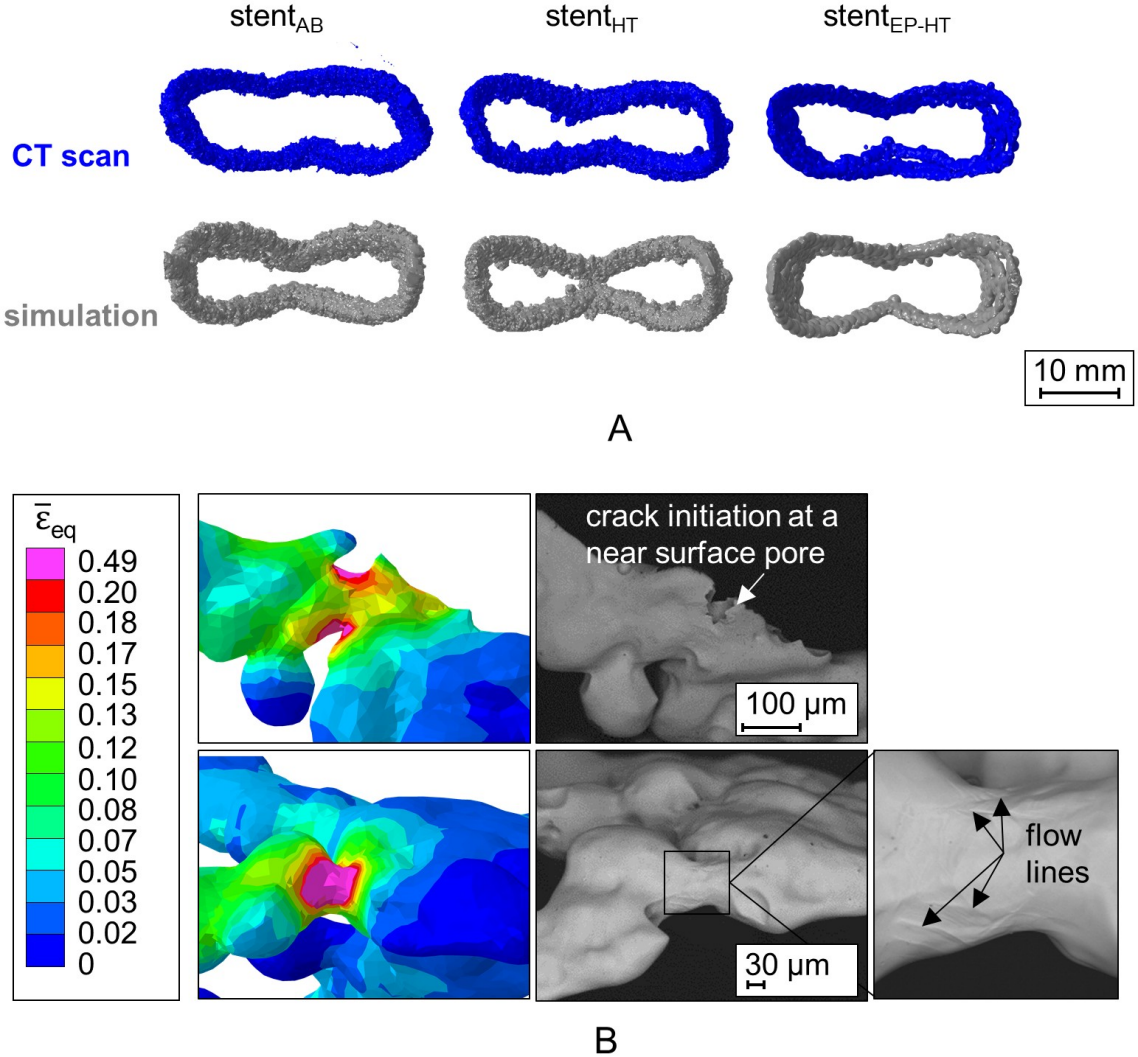
Qualitative validation of the uniaxial stent compression simulations. A: Comparison of the numerically predicted deformed stent shape (gray) with the X-ray CT reconstructions of the corresponding experimentally compressed laser powder bed fused (L-PBF) stent (blue). B: Comparison of scanning electron microscopy images with the equivalent plastic strain ${\bar{\epsilon }}_{\text{eq}}$ in areas of large deformations for stent_EP-HT_, near a surface crack (top row) and near strut nodes (bottom row). Stent_AB_, stent_HT_ and stent_EP-HT_ correspond to the reconstructed L-PBF in the as-built (AB), heat treated (HT), and electropolished and HT (EP-HT) conditions, respectively.

For a further qualitative validation of the stent compression analysis, regions of the deformed stent models are compared to corresponding regions of the experimentally deformed stents images using both X-ray CT and SEM (Fig 13). Based on the X-ray CT reconstructions (Fig 13A), good agreement is observed between the numerically predicted and the experimentally compressed stent configurations. Minor deviations are attributed to deviations in the initial stent position between the plates or a slight side movement of the stent at the beginning of the compression. Furthermore, particularly deformed areas are identified using SEM and compared with the equivalent plastic strain ${\bar{\epsilon }}_{\text{eq}}$ distribution determined in the simulation (Fig 13B). Areas of large plastic deformation can be identified in SEM images by means of so-called flow lines indicated by a wavy surface near local crack formation, e.g. due to a pore close to the surface, or near nodes (connecting points of the struts). A good agreement between the predicted high local plastic deformation with a crack and with local flow lines is observed. Thus, the results of this qualitative validation suggest that the adjustment of the material properties (Fig 11A) is appropriate.

#### 3.3.3 Stress distribution within the laser powder bed fused stent during compression

**Fig 14:**
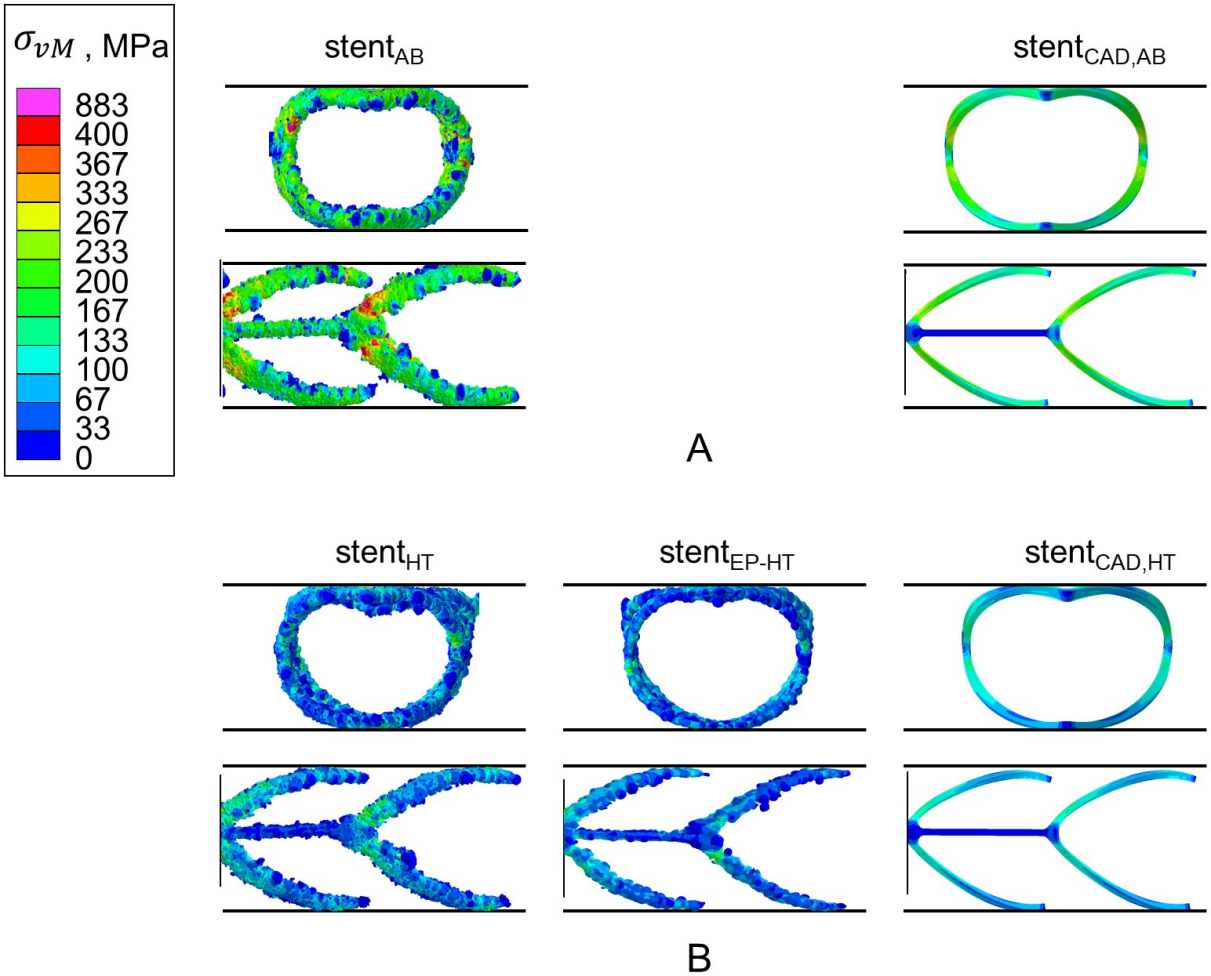
Contour plots of the von Mises stress *σ*_vM_ on the outer surface of the laser powder bed fused (L-PBF) stents under compression of 0.8 mm. A: Stents in the as-built and B: in the heat treated material condition illustrated in the front (top row) and the side view (bottom row). Stent_AB_, stent_HT_ and stent_EP-HT_ correspond to the reconstructed L-PBF stents in the as-built (AB), heat treated (HT), and electropolished and HT (EP-HT) conditions, respectively. Stent_CAD,AB_ and stent_CAD,HT_ correspond to the as-designed stent CAD models with the as-built and heat treated material properties, respectively.

In terms of the von Mises equivalent stress distribution *σ*_vM_, a significant reduction of *σ*_vM_ is observed for the heat treated (Fig 14B) compared to the as-built (Fig 14A) stents. This trend is not surprising given the relation of the flow curves for the as-built and heat treated material condition (Fig 11A). Due to the geometric irregularities, local stress peaks occur in the actual L-PBF stents with maximum stresses *σ*_vM_ of 883 MPa for stent_AB_, 813 MPa for stent_HT_ and 341 MPa for stent_EP-HT_. Although, due to the small structural size electropolishing cannot fully compensate for pre-existing strut waviness and tapers, it does serve to level out or round local protrusions. The smoothed surface therefore results in the observed reduced local stress concentrations that develop during stent deformation and thus can mitigate the formation of local damage/cracks. Studies on electropolished L-PBF 316L have also shown that smoothing the surface of L-PBF 316L increases fatigue life [56].

Although, electropolishing has been found to reduce stress concentration by reducing the impact of local process-related geometrical irregularities, it can cause local weakening of the stent or further reduce the local strut diameter in regions of pre-existing (process-induced) reductions. In other words, although the electropolishing process is effective at removing large protruding features, it can also remove material from already reduced strut diameters, making this defect even more critical. In addition, near-surface defects such as pores can become exposed, which then act as surface notches during stent deformation (Fig 13B). It is therefore essential to determine appropriate parameters for an effective electropolishing and to check the stents for exposed defects or drastically reduced strut cross sections after surface treatment. The electropolishing process should be adjusted to ensure surface treatment of all areas of the stent. In addition, the advantages and disadvantages of near-net-shape stent fabrication with subsequent low ablation by electropolishing should be compared to oversized stent fabrication with subsequent high ablation by electropolishing.

In terms of the von Mises equivalent stress distribution *σ*_vM_, the L-PBF stents show a spatial variability of stress, while the CAD model (stent_CAD_) has a smooth and more constant stress distribution (Fig 14). At local geometric irregularities, such as notches and regions of reduced diameters, local stress peaks are observed in the L-PBF stents, in contrast to the CAD model. In the as-built material condition, the maximum stress for the stent_AB_ is 883 MPa; whereas, the CAD model stent_*CAD*, *AB*_ has a maximum value of 312 MPa. The maximum stresses for the heat treated L-PBF stent without (stent_HT_) and with (stent_EP-HT_) surface treatment are 813 MPa and 341 MPa, respectively; whereas, the CAD model stent_*CAD*, *HT*_ has a maximum value of 180 MPa.

### 3.4 Numerical analysis of the expansion behavior of laser powder bed fused stents

#### 3.4.1 Global deformation behavior of laser powder bed fused stent during expansion

**Fig 15:**
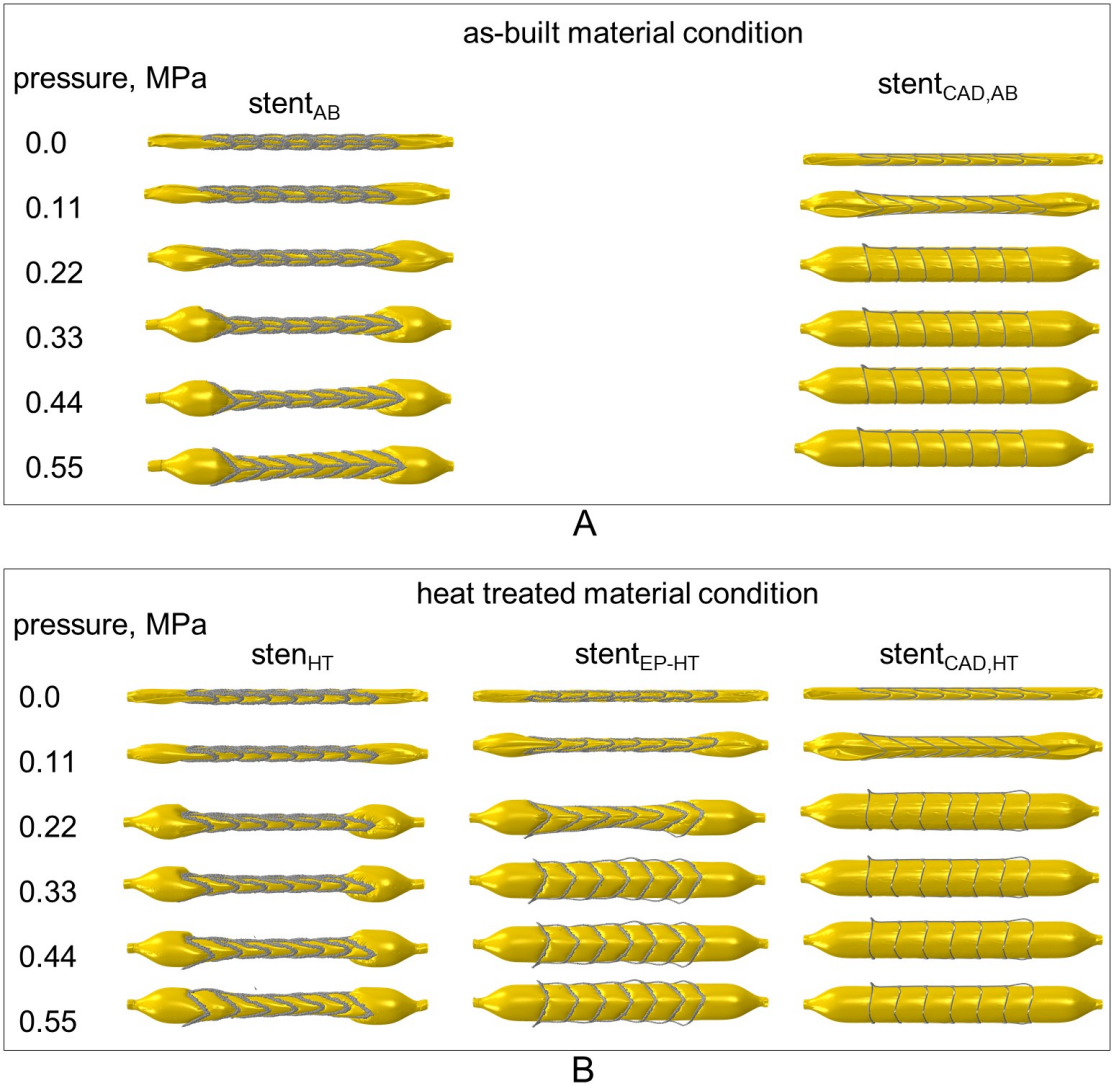
Predicted global deformation behavior of laser powder bed fused (L-PBF) stents during expansion. A: Predicted expansion behavior of L-PBF stent in the as-built material condition. B: Predicted expansion behavior of L-PBF stent in the heat treated material conditions. Stent_AB_, stent_HT_ and stent_EP-HT_ correspond to the reconstructed L-PBF stents in the as-built (AB), heat treated (HT), and electropolished and HT (EP-HT) conditions, respectively.

The expansion behavior of the L-PBF stents is shown in Fig 15. Similar to stent compression, the geometrically induced stiffness of the L-PBF stents has a great influence on their expandability and, consequently, on safety during expansion.

The stents without surface treatment (stent_AB_, stent_HT_) have high stiffness and thus exhibit high resistance to balloon expansion. Despite very high pressure accumulation at the free balloon ends, the initial expansion of the stent ends is hardly present for the stents without surface conditioning (stent_AB_, stent_HT_). This pressure accumulation, in combination with the surface roughness of the L-PBF stents, which could potentially weaken the balloon during crimping due to protruding features, poses a potential risk for the balloon to burst. These effects can be largely mitigated through the use of electropolishing, which is discussed below. A relatively homogeneous diameter increase over the entire stent length is observed for these stents starting at an expansion pressure of approximately 0.55 MPa.

The predicted expansion behavior of the electropolished and heat treated stent (stent_EP-HT_), corresponds most closely to the inhomogeneous expansion behavior of conventional stents under the development of the so-called dogbone effect [33] (S4 Fig). Thereby, the crimped stent provides resistance to expansion, which causes the expansion pressure to accumulate at the free balloon ends. After exceeding a certain threshold value, the pressure is sufficient to expand the ends of the stent, making the stent resemble a dogbone. The stent is then expanded towards its center as if by a wedge until the final cylindrical shape is reached. Besides reducing the accumulation of pressure at the balloon ends, electropolishing also reduces the risk of local balloon damage with the smoothing of the strut surface.

Similar to the compression analysis, significant differences between the expansion behavior of the reconstructed L-PBF stents and as-designed CAD stents are observed. Due to its lower mass, the CAD stent has a lower radial stiffness and therefore offers less resistance to balloon expansion. Therefore, in contrast to L-PBF stents without surface treatment (stent_AB_, stent_HT_), no excessive pressure accumulation occurs at the free balloon ends during the expansion of the CAD stent. The CAD stent is also less stiff than stent_EP-HT_, so the dogbone effect is less pronounced (Fig 15). Further, full stent expansion occurs at half the expansion pressure as that for stent_EP-HT_ and thus resulting in an underestimation of the necessary expansion pressure. With regard to the final expanded shape, stent_EP-HT_ shows buckled stent struts, while the idealized stent has all struts properly conformed to the balloon contour.

#### 3.4.2 Stress distribution within the laser powder bed fused stents during expansion

**Fig 16:**
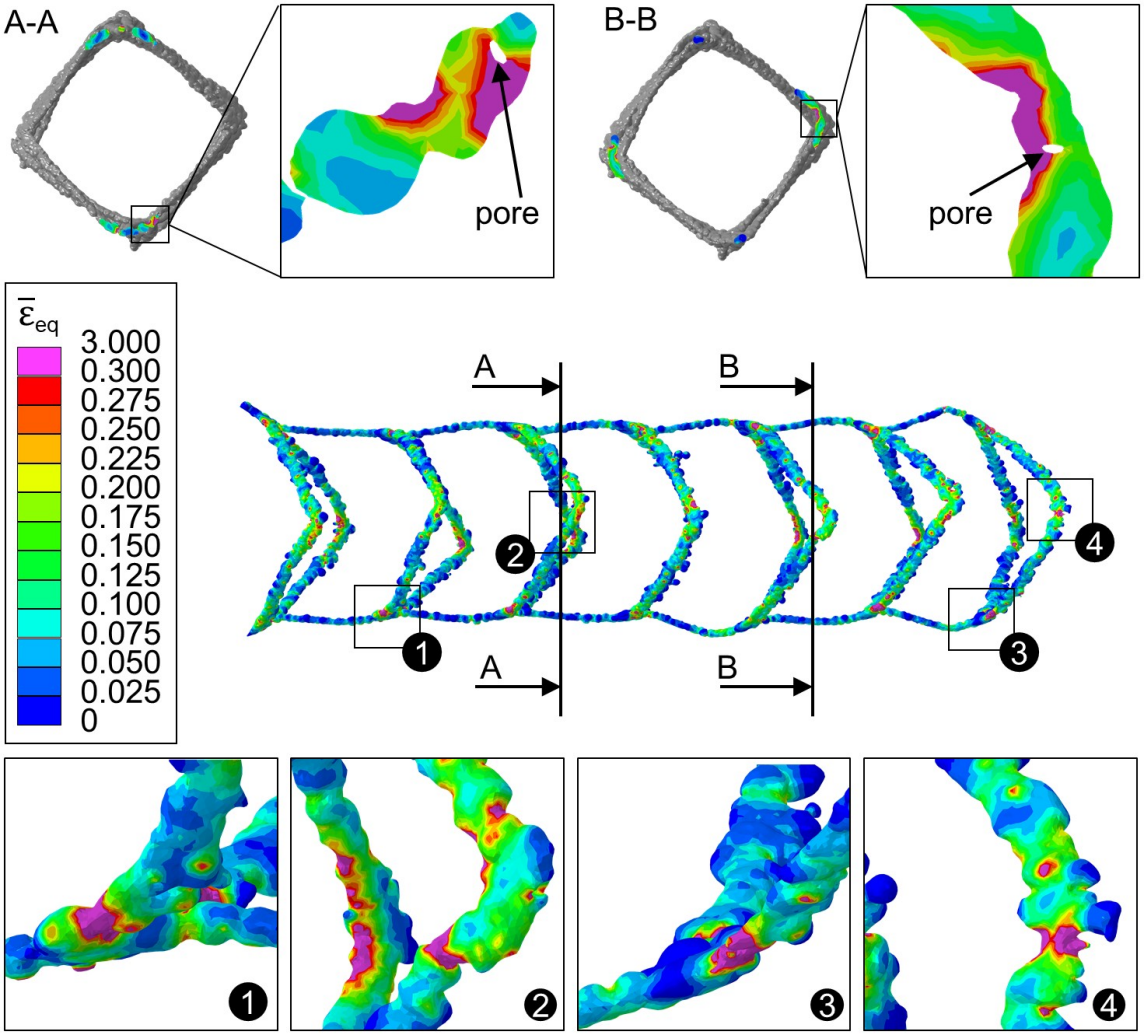
Influence of reduced strut diameters/notches and pores (section A-A, B-B) for the electropolished and heat treated stent (stent_EP-HT_) in the area of large plastic deformation using the contour plot of equivalent plastic strain ${\bar{\epsilon }}_{\text{eq}}$ distribution.

The von Mises stress (*σ*_vM_) distribution shows the same trend as that discussed in the compression analysis (Fig 14); thus, it is not explicitly visualized for each stent. Due to the geometric irregularities, the stresses are generally elevated and local stress peaks occur in the actual L-PBF stents compared to the CAD stent. Heat treatment again leads to a significant reduction of the stresses. The influence of geometric irregularities such as notches, locally reduced diameters and pores on the development of local strain concentrations is exemplary demonstrated using the equivalent plastic strain ${\bar{\epsilon }}_{\text{eq}}$ distribution within stent_EP-HT_ (Fig 16). In the expanded state, high strains occur preferably at locally reduced strut diameters/notches and at the stent spikes. The equivalent plastic strain ${\bar{\epsilon }}_{\text{eq}}$ locally exceeds 30%, which corresponds to the uniform elongation of a conventional 316L and can therefore be associated with the onset of local damage. Pores within the struts lead to a local reduction of the effective strut cross section, which is critical in areas that are expected to undergo large plastic deformation (Fig 16, sectional drawing A-A, B-B). Due to their strength-reducing effect, pores and notches and in particular their superposition should be considered critical, as theses could lead to early local failure. This is particularly critical in regions subjected to large deformations and especially for structures with a high surface-to-volume ratio. For stents, the transition area between the nodes and the struts, and the stent spikes, are particularly susceptible. The strength-reducing effect of these geometric irregularities can pose risks not only during the implantation process, but also during the long-term usage of the stent under the cyclic loading caused by changes in the blood pressure. A study on the influence of internal porosity and surface roughness on the fatigue behavior of L-PBF 316L round tensile specimens (4.5 cm diameter) has shown that at low load levels and a high number of load cycles to failure (high-cycle fatigue), damage initiates mainly at the surface; whereas, at high load levels and low number of cycles (low-cycle fatigue), damage is increasingly initiated by internal pores [43]. Transferred to the life-cycle of L-PBF stents, the impact of pores during the implantation process and the impact of surface roughness over the long-term application must, therefore, be regarded as critical. Further investigations are necessary to quantify the impact of these geometric irregularities on the damage and fatigue behavior of L-PBF stents.

Regarding the von Mises stress (*σ*_*vM*_) distribution, the comparison of the stress distribution within the CAD model (stent_CAD_) with the actual L-PBF stents shows same trend as described in the compression analysis. Therefore, due to the geometric irregularities, the stresses are generally elevated and local stress peaks occur in the actual L-PBF stents compared to the CAD stent.

### 3.5 Limitations and scope for future research

Stent manufacturing via L-PBF presents a major challenge. In this study, L-PBF stents with a strut thickness of 200 *μ*m could be realized using a conventional commercial L-PBF machine, however, with a variety of geometrical imperfections. In a follow-up study, the challenges identified here will be addressed by using a micro-L-PBF machine with a smaller laser spot size, a different recoating strategy, a smaller powder particle size and a related comprehensive process parameter study, as proposed in Ref. [57].

Furthermore, the inverse determination of the material properties of the stent should be discussed. The determination of material properties on micro-specimens has been omitted in this study due to significant challenges associated with specimen preparation (e.g large aspect ratios between strut diameter and length), experimental testing (e.g. clamping, inaccuracy of strain measurement), and determination of specimen dimensions (e.g. determination of specimen cross section) [54]. In the literature, data are available for L-PBF 316L struts in the as-built state, but not in the heat treated condition [53, 54, 58]. Therefore, for consistency reasons, the flow curves were inversely determined from the experimentally determined global response of the stent under compression using FEA. The verification of the flow curves with literature data (as-built condition)and the implementation of the determined flow curve in the FEA of a further stent morphology (stent_EP-HT_, heat treated condition) show that this approach is justified (Figs 11B and 12). In a follow-up study, the material properties of the L-PBF stents will be determined by means of comprehensive experiments on micro-specimens, together with the optimization of the L-PBF process. This will improve the accuracy of the method for numerical analysis of the L-PBF stents presented here.

In this study, heat treatment was primarily used to increase the deformability of the stents to enable their crimping and expansion. Studies in literature have found that the corrosion resistance of L-PBF 316L is inferior to that of wrought 316L, but also that it improved by heat treatment [52]. Since the corrosion resistance is essential biocompatibility of the stents, further studies are necessary to improve the corrosion behavior of L-PBF 316L stents by adequate heat treatment.

There are a number of modeling assumptions made in this work that merit further discussion. In addition to assumptions made regarding the boundary conditions and the material model, the accuracy of the FEA results is mainly influenced by the fidelity of the morphological representation of the investigated structure. The L-PBF stent morphologies are based on reconstruction from X-ray CT data with a voxel size of 8.5 *μ*m. In general, a minimum width of three voxels is required for structures to be reliably captured by CT [59]. Therefore, only pores and adherent powder particles with a diameter of more than 25.5 *μ*m are considered within this study (S5 Fig). Adhering powder particles are either neglected based on the aforementioned size threshold or are modeled as completely fused to the strut surface, making them load-bearing, in contrast to reality. The latter modeling assumption artificially increases the local strut thickness. Combined with the omission of very small pores, this can lead to a possible increase in global stent mass stiffness. Therefore, the inversely determined yield strength of the material comprising the L-PBF 316L stent might slightly underestimate the actual value to compensate for the artificially increased predicted compression strength.

The L-PBF process is also associated with an anisotropic material behavior. In this study, the material behavior of the stent was considered isotropic and homogeneous assuming that the geometric effects (e.g. stent design, irregularities) outweigh the influence of material anisotropy with respect to the macroscopic mechanical behavior [27]. Although this approach is well established for L-PBF lattice structures [19, 54, 60, 61], the direction-dependent properties of the stent should be further investigated (e.g. influence of the strut orientation to the build direction) and should be considered within the material description.

The aim of this study was not to determine the exact material properties of L-PBF 316L stents. However, this work has shown that the size effect has a significant influence on the mechanical properties of L-PBF 316L and therefore has to be considered for the design of filigree L-PBF structures. Furthermore, a methodology is presented that enables the determination of the material properties of L-PBF stents and thus allows to investigate their mechanical behavior more accurately within FEAs. Besides the size effect, the mechanical properties of L-PBF metals are influenced by a variety of factors such as powder or L-PBF process parameters, which causes variations in the mechanical properties of the same component in different batches.

In this study, heat and surface treatment has been found to be critical factor for the feasibility of stents expansion. Therefore, further experimental studies are required to identify adequate heat and surface treatments. For L-PBF metals, heat treatment is further associated with an increase in fatigue properties [46]. However, as damage and fatigue properties are strongly influenced by surface roughness, internal defects, and structural size, further numerical and experimental studies are required to make more quantitative statements regarding the long-term performance of L-PBF stents. In this context, the biocompatibility of L-PBF 316L should also be investigated more closely.

It has also been shown that the numerical analysis of L-PBF stents based on the as-designed CAD model is not suitable in its current form. Since numerical stent analysis based on reconstructed CT data is very complex and computationally intensive, a method for the creation of synthetic stents based on statistical methods, e.g. as described in Ref. [19] for L-PBF lattice structures, should be developed.

## 4 Conclusions

In this study, the impact of L-PBF process-related geometrical irregularities, the size effect, heat treatment, and surface treatment on the mechanical response of L-PBF 316L stents was investigated using a combined experimental and computational framework based on the reconstruction of actual L-PBF stents. The following conclusions are drawn:

1. Considering the extent of process-related geometric irregularities (e.g., strut waviness, pores, notches, deviations in strut diameter,) is essential for the development and evaluation of L-PBF stents. Such irregularities can lead to significant deviations in the morphology and thus the mechanical properties of L-PBF stents compared to as-designed (idealized) stents.
2. L-PBF process-related geometric irregularities lead to a significant increase in the strut diameter and thus in the geometric resistance of the actual L-PBF stents, which can be partially reduced by surface treatment such as electropolishing.
3. Surface treatment (viz., electropolishing) of L-PBF stents is essential to achieve a radial compression strength and thus a stent expansion behavior comparable to that of conventional stents. Proper electropolishing serves to smooth the strut surface, reduce excessive material volume caused by the L-PBF process, reduce stent stiffness, and increase global deformability, thus reducing the risk of balloon damage/bursting during expansion.
4. Adequate heat treatment of the L-PBF stent is essential to improve the ductility of the stent, to reduce global stress and, in combination with surface treatment, to reduce local stress concentrations, which could help to mitigate the risk of stent damage during implantation and cyclic loading.
5. The size effect has a significant influence on the material properties of L-PBF 316L, manifested by a reduction in the yield strength of material comprising filigree L-PBF 316L structures by 41% in the as-built and by 59% in the heat treated condition compared to bulk material. This size effect should be considered for accurate prediction of L-PBF stent behavior.
6. For a numerical analysis of L-PBF stents a reconstruction or statistical consideration of process-related irregularities is essential. Otherwise, the radial stiffness of the stents is underestimated and possible local stress concentrations are neglected.
7. To further improve stent morphology and mechanical behavior, further studies with dedicated micro-L-PBF machines and comprehensive process parameter studies are required.

This work marks an important step toward establishing L-PBF as a viable route for manufacturing patient-specific cardiovascular stents. The findings from this work motivate future experimental/numerical studies to quantify threshold values of critical geometric irregularities, which could be used to establish design guidelines for L-PBF stents/lattice structures. Such studies could include identification of the maximum allowable degree of porosity or surface waviness in relation to strut thickness. Additionally, further experimental investigations are needed to identify optimal parameters for the manufacturing process, heat treatment, and surface treatment for L-PBF stents to establish the application of L-PBF in the field of cardiovascular stents.

## Supporting information

S1 Fig Stress-strain curves of the flat tensile specimens with a specimen thickness. The flat tensile specimens had a thickness of 1 mm and specimen orientation of 0° to build direction. AB refers to the tensile specimen in the as-built condition and HT to the tensile specimen in the heat treated condition, respectively.
(TIF)

S2 Fig Mesh convergence study. A: Impact of mesh refinement on the macroscopic response of an laser powder bed fused (L-PBF) stent under compression. B: Impact of mesh refinement on the radial force at a compression of 1.4 mm with respect to the number of elements. C: Magnified view of the mesh of an L-PBF stent at the respective mesh refinement.
(TIF)

S3 Fig Mechanical response of laser powder bed fused (L-PBF) stents using the initial flow curves determined from tensile tests. Stent_AB_, stent_HT_ and stent_EP-HT_ correspond to the reconstructed L-PBF stents in the as-built (AB), heat treated (HT), and electropolished and HT (EP-HT) conditions, respectively. The experimental determined response of the selectively laser melted stents is illustrated by the shaded curve areas.
(TIF)

S4 Fig Comparison of the expansion behavior of a laser powder bed fused (L-PBF) and a conventional laser-cut stent. A: Predicted expansion behavior of stent_EP-HT_ based on the the reconstruction of an electropolished and heat treated L-PBF stent from X-ray CT data. B: Numerical predicted (left) and experimental determined expansion behavior of an conventional laser-cut stent [33].
(TIF)

S5 Fig Effects of the CT resolution on the accuracy of the numerical analysis. A: Comparison of the strut morphology of an laser powder bed fused stent (as-built condition) reconstructed on the basis of CT data with scanning electron microscopy images. B: Illustration of the limited resolution of the internal porosity (effective pore diameter *D*_*Pore*_ > 25.5 *μ*m).
(TIF)

S6 Fig Experimental determined mechanical response of laser powder bed fused stents under compression. The as-built (AB) stents are represented by the black lines, the heat treated stents (HT) by the red lines and the electropolished and heat treated (EP-HT) stents by the blue lines.
(TIF)

S1 Table Experimental determined radial force at 50% compression F_rad. 50%_ and the corresponding mass m of the respective stent configuration. (PDF)
