# Supplementary figures and images for: Computational analysis of the effects of geometric irregularities and post-processing steps on the mechanical behavior of additively manufactured 316L stainless steel stents
Source: PLoS One. 2020 Dec 29;15(12):e0244463. doi: 10.1371/journal.pone.0244463 (PMC7771678; doi:10.1371/journal.pone.0244463)

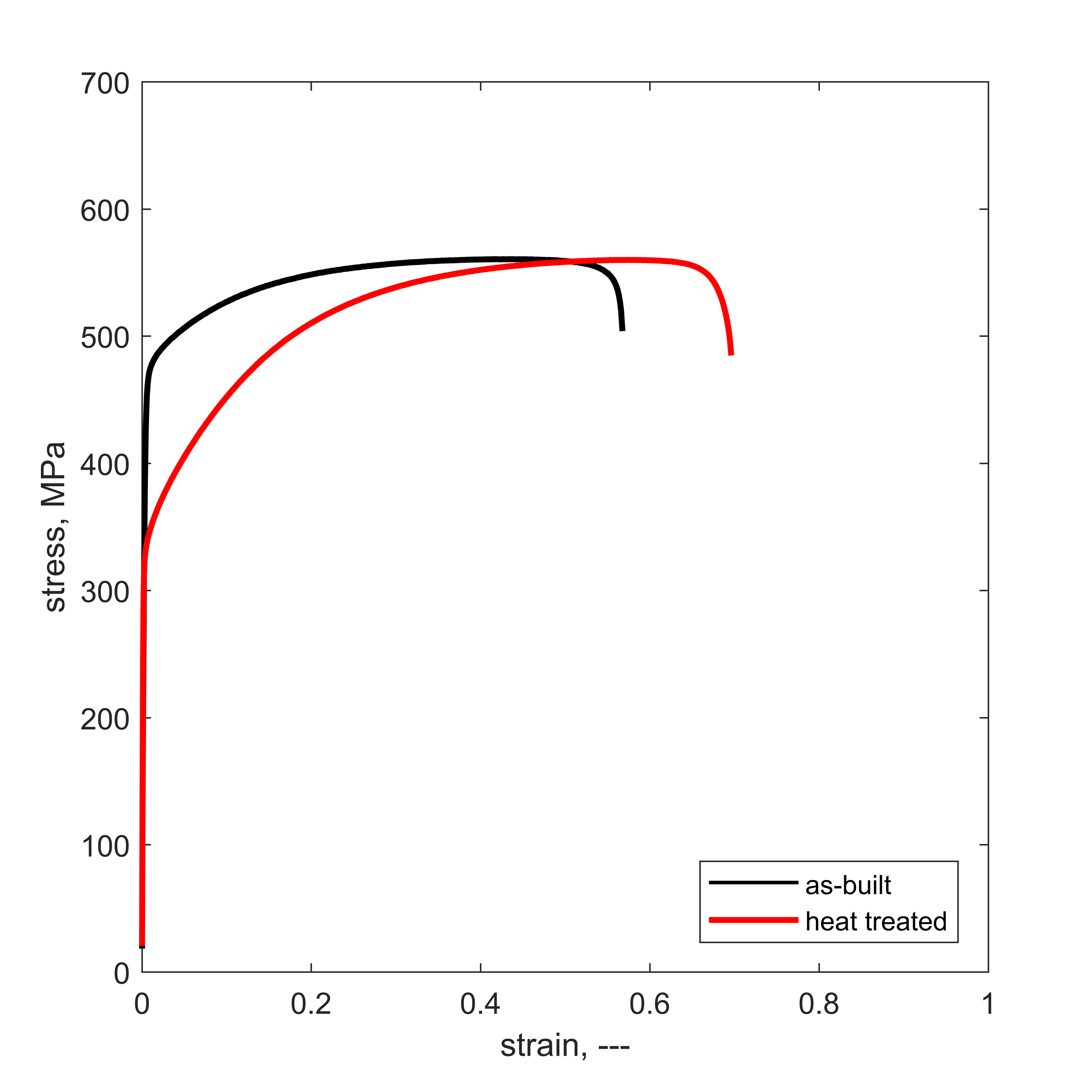

Supplement: S1 Fig — The flat tensile specimens had a thickness of 1 mm and specimen orientation of 0° to build direction. AB refers to the tensile specimen in the as-built condition and HT to the tensile specimen in the heat treated condition, respectively. (TIF) [file pone.0244463.s001.tif]

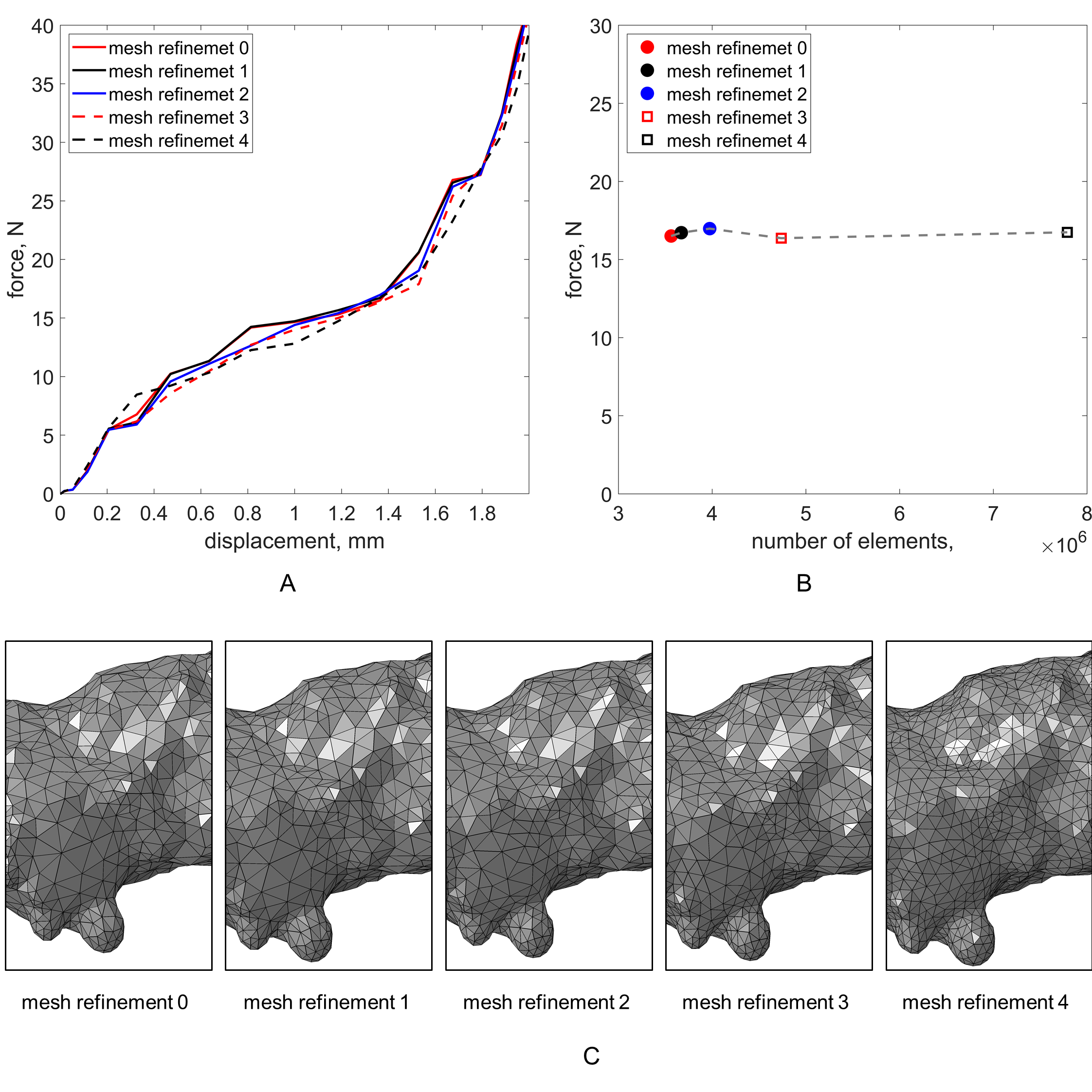

Supplement: S2 Fig — A: Impact of mesh refinement on the macroscopic response of an laser powder bed fused (L-PBF) stent under compression. B: Impact of mesh refinement on the radial force at a compression of 1.4 mm with respect to the number of elements. C: Magnified view of the mesh of an L-PBF stent at the respective mesh refinement. (TIF) [file pone.0244463.s002.tif]

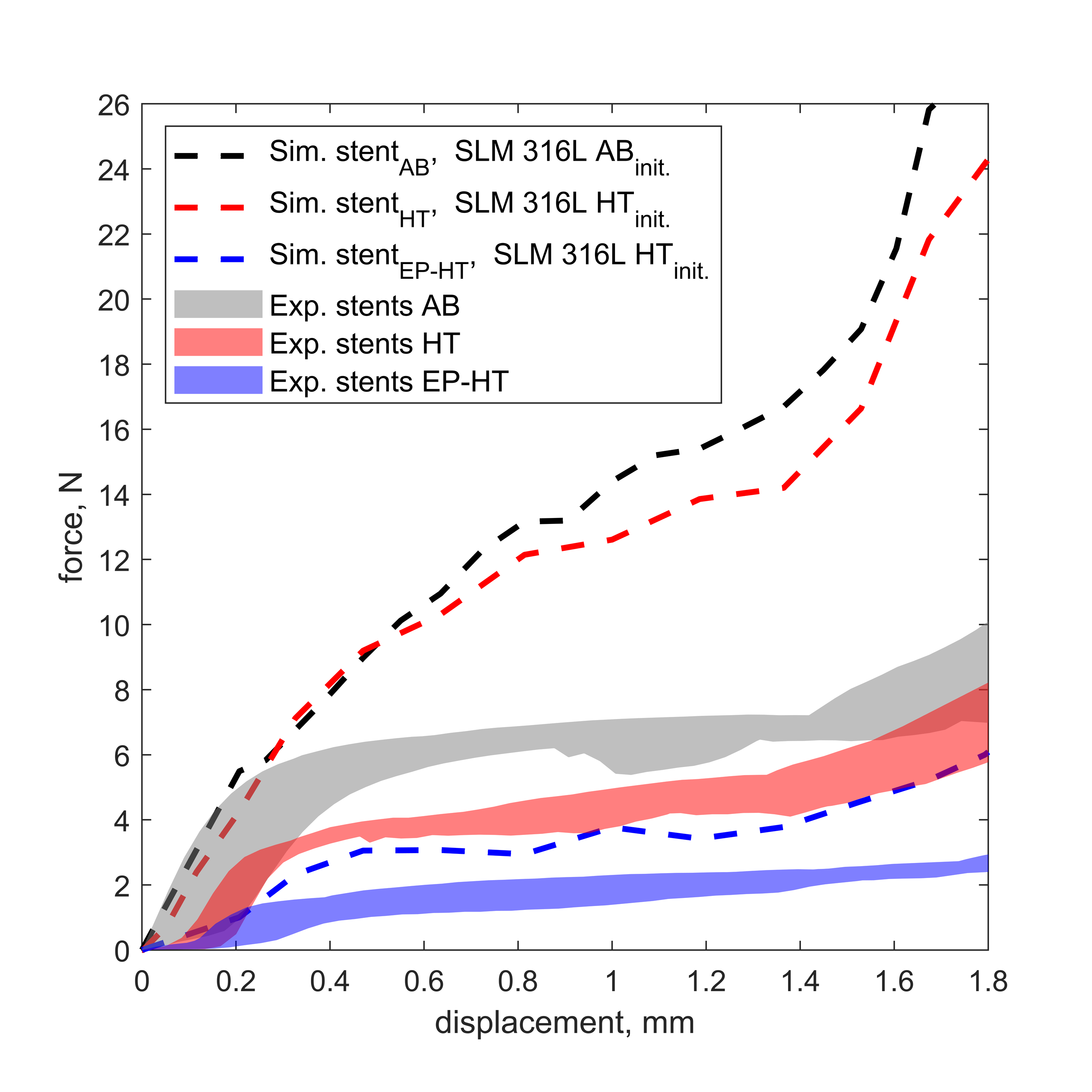

Supplement: S3 Fig — StentAB, stentHT and stentEP-HT correspond to the reconstructed L-PBF stents in the as-built (AB), heat treated (HT), and electropolished and HT (EP-HT) conditions, respectively. The experimental determined response of the selectively laser melted stents is illustrated by the shaded curve areas. (TIF) [file pone.0244463.s003.tif]

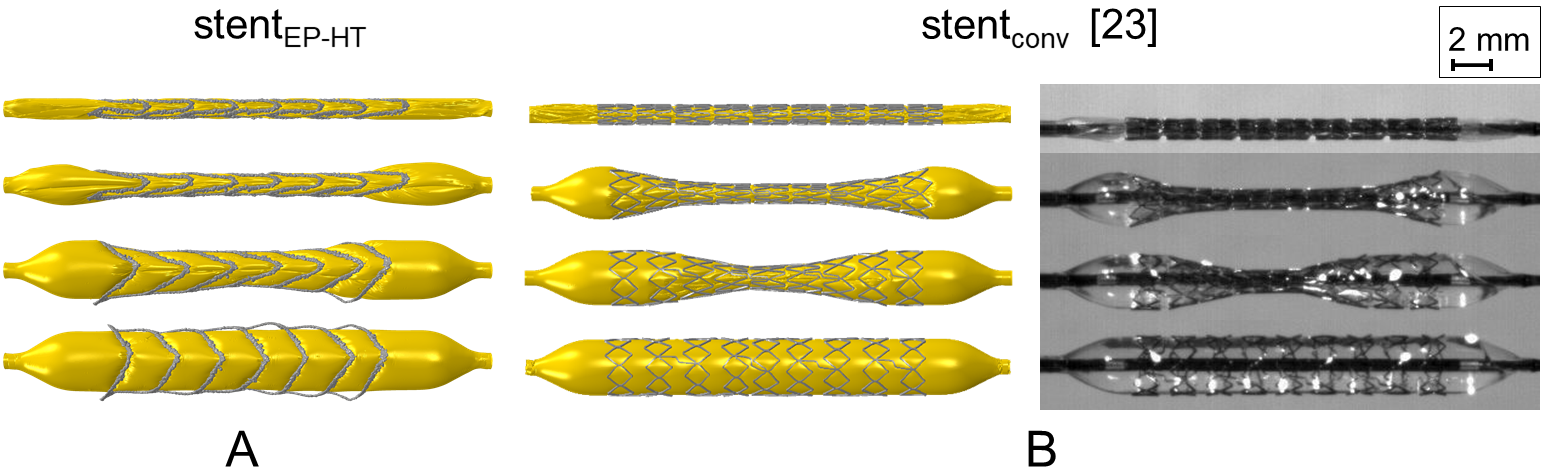

Supplement: S4 Fig — A: Predicted expansion behavior of stentEP-HT based on the the reconstruction of an electropolished and heat treated L-PBF stent from X-ray CT data. B: Numerical predicted (left) and experimental determined expansion behavior of an conventional laser-cut stent [33]. (TIF) [file pone.0244463.s004.tif]

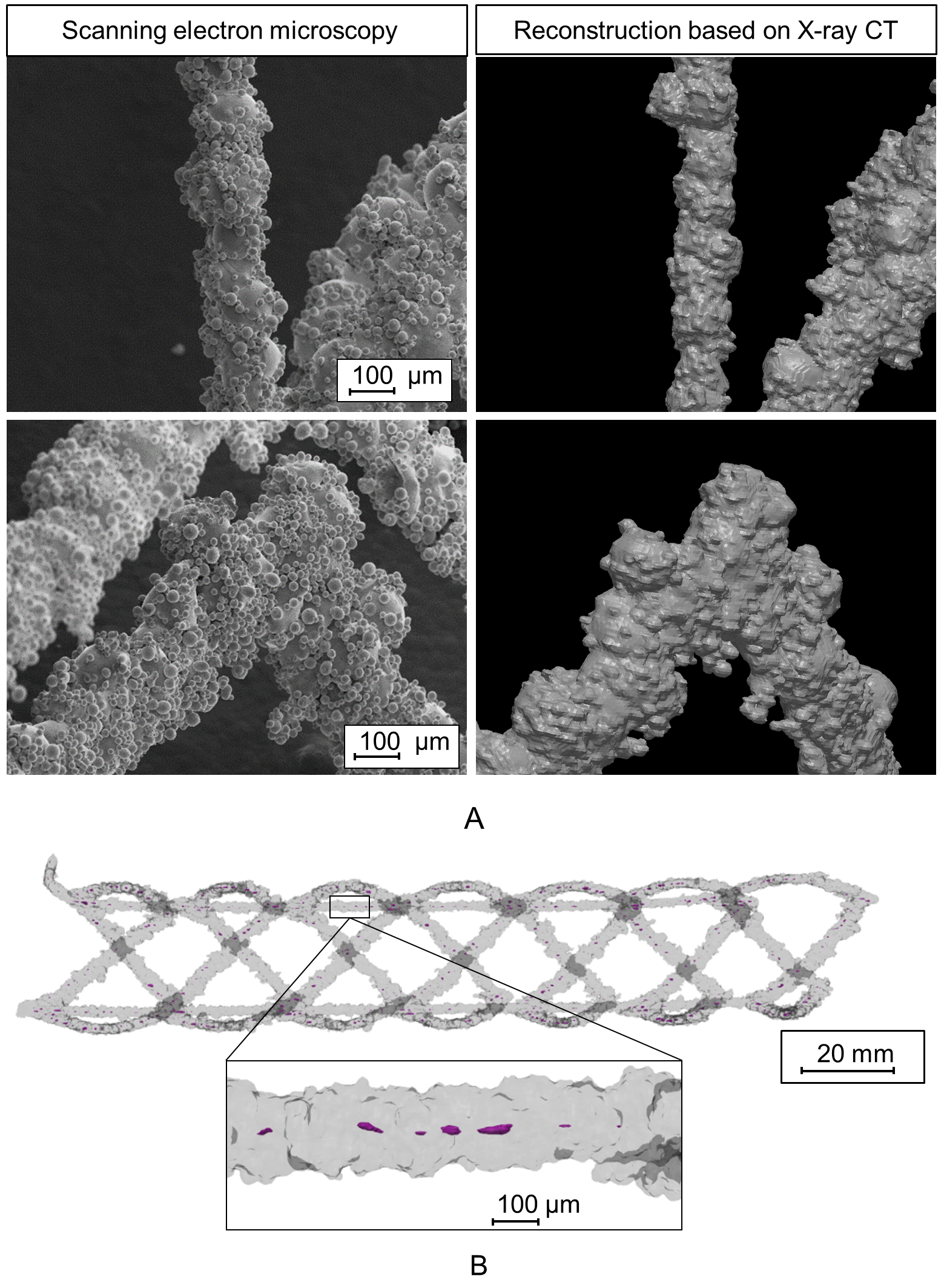

Supplement: S5 Fig — A: Comparison of the strut morphology of an laser powder bed fused stent (as-built condition) reconstructed on the basis of CT data with scanning electron microscopy images. B: Illustration of the limited resolution of the internal porosity (effective pore diameter DPore > 25.5 μm). (TIF) [file pone.0244463.s005.tif]

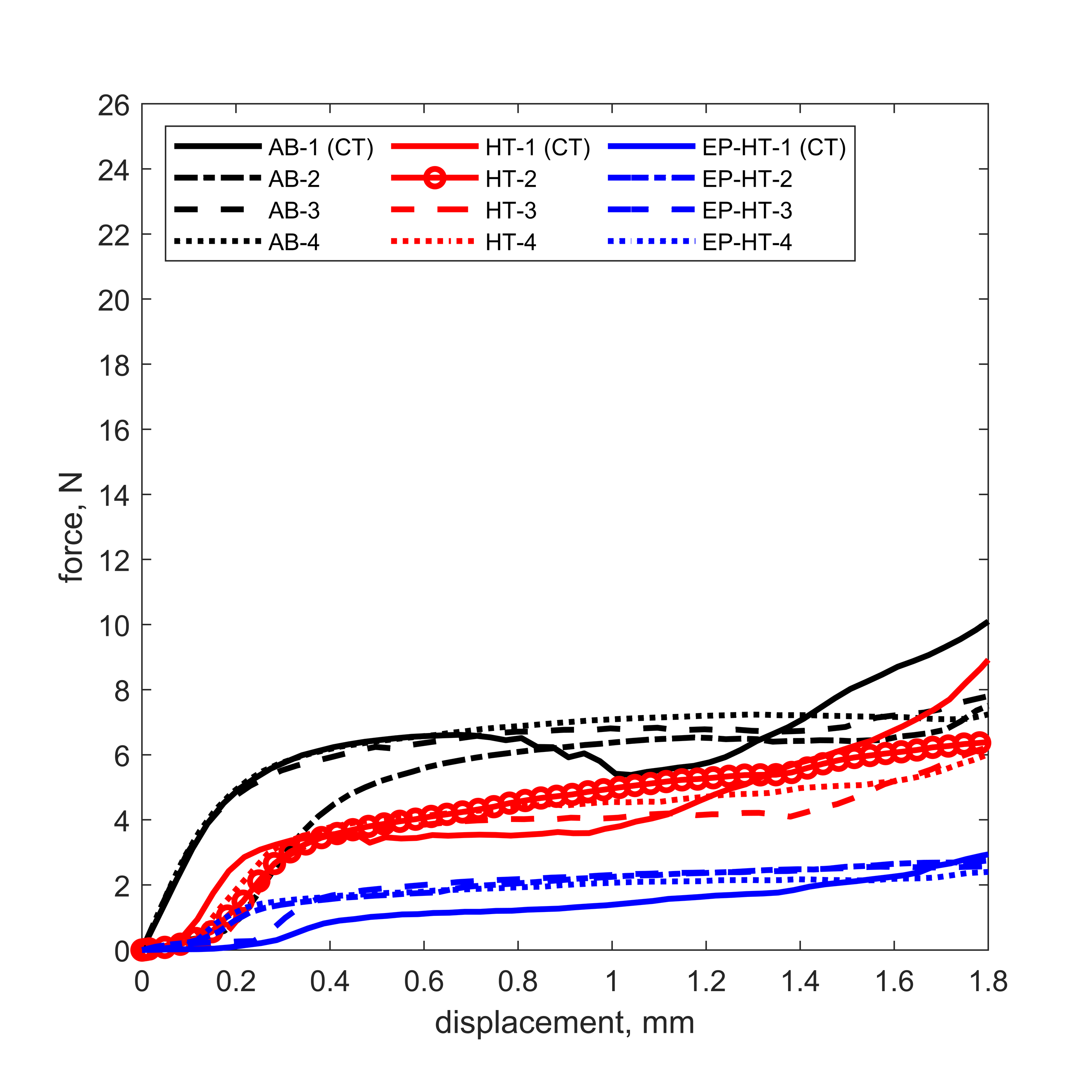

Supplement: S6 Fig — The as-built (AB) stents are represented by the black lines, the heat treated stents (HT) by the red lines and the electropolished and heat treated (EP-HT) stents by the blue lines. (TIF) [file pone.0244463.s006.tif]
