# Supplementary material for: Computational analysis of the effects of geometric irregularities and post-processing steps on the mechanical behavior of additively manufactured 316L stainless steel stents
Source: PLoS One. 2020 Dec 29;15(12):e0244463. doi: 10.1371/journal.pone.0244463 (PMC7771678; doi:10.1371/journal.pone.0244463)
Supplement: S1 Table — (PDF) [file pone.0244463.s007.pdf]

S1 Table. Experimental determined radial force at 50% compression $F_{rad.\ 50\%}$ and the corresponding mass m of the respective stent configuration

Stent configuration	$F_{rad.\ 50\%}$, N	m, g
Stent AB-1 (CT, $stent_{AB}$)	7.74	0.0412
Stent AB-2	6.41	0.0422
Stent AB-3	6.90	0.0435
Stent AB-4	7.19	0.0422
Stent HT-1 (CT, $stent_{HT}$)	5.92	0.0421
Stent HT-2	6.28	0.0429
Stent HT-3	4.66	0.0411
Stent HT-4	5.06	0.0423
Stent EP-HT-1 (CT, $stent_{EP\text{-}HT}$)	2.52	0.0223
Stent EP-HT-2	2.55	0.0252
Stent EP-HT-3	2.51	0.0257
Stent EP-HT-4	2.56	0.0248

AB, HT and EP-HT referees to the as-built, heat treated, and electropolished and heat treated condition, respectively. The stents used for the model reconstruction of the laser powder bed fused stent models are marked by the reference (CT, $stent_{XX}$).
